# A Double Team Semantics for Generalized Quantifiers

**DOI:** 10.1007/s10849-015-9217-4

**Published:** 2015-04-28

**Authors:** Antti Kuusisto

**Affiliations:** Institute of Computer Science, University of Wrocław, ul. Joliot-Curie 15, 50-383 Wroclaw, Poland; Department of Philosophy, Stockholm University, 10-691 Stockholm, Sweden

**Keywords:** Team semantics, Dependence logic, Generalized quantifiers, Game-theoretic semantics

## Abstract

We investigate extensions of dependence logic with generalized quantifiers. We also introduce and investigate the notion of a *generalized atom*. We define a system of semantics that can accommodate variants of dependence logic, possibly extended with generalized quantifiers and generalized atoms, under the same umbrella framework. The semantics is based on pairs of teams, or *double teams*. We also devise a game-theoretic semantics equivalent to the double team semantics. We make use of the double team semantics by defining a logic $\hbox {DC}^2$ which canonically fuses together *two-variable dependence logic*$\hbox {D}^2$ and *two-variable logic with counting quantifiers*$\hbox {FOC}^2$. We establish that the satisfiability and finite satisfiability problems of $\hbox {DC}^2$ are complete for $\hbox {NEXPTIME}$.

## Introduction

*Independence-friendly logic* is an extension of first-order logic motivated by issues concerning Henkin quantifiers and game-theoretic semantics. Independence-friendly logic, also known as IF-logic, was first defined by Hintikka and Sandu (1989). The logic extends first-order logic FO by quantifiers of type $\exists x/\{y_1,\ldots ,y_k\}$. The background intuition concerning the interpretion of these quantifiers is that when a formula $\exists x/\{y_1,\ldots ,y_k\}\, \varphi$ is evaluated game-theoretically, then the value of *x* is chosen in ignorance of the values of the variables $y_1,\ldots ,y_k$.

While game-theoretic semantics of ordinary first-order logic gives rise to a game of perfect information, the game for IF-logic is a game of imperfect information. Hodges (1997) gave a compositional semantics for IF-logic. While ordinary Tarskian semantics for first-order logic is based on evaluating formulae with respect to single *assignments*,^1^ the semantics of Hodges is based on *sets* of assignments.

Väänänen (2007) introduced *dependence logic*, which provides a novel alternative approach to issues concerning independence-friendly logic and Henkin quantifiers. Instead of quantifiers of type $\exists x/\{y_1,\ldots ,y_k\}$, dependence logic extends first-order logic by novel atomic expressions $=\!\!(x_1,\ldots ,x_k)$ which state that the value of $x_k$ is functionally determined by the values of $x_1,\ldots ,x_{k-1}$. The compositional semantics of dependence logic is similar to Hodges’ semantics for IF-logic. The semantics is formulated in terms of sets of assignments. Väänänen named such sets *teams*, and since then, the related semantic framework has been called *team semantics*.

After the introduction of dependence logic, research on team semantics has been very active, and a notably large number of related papers has appeared in the course of a relatively short period. In addition to dependence logic, several related logics have been introduced and studied.

*Independence logic*, introduced by Grädel and Väänänen (2013), extends first-order logic with atoms of $x\, \bot \, y$. The intuitive meaning of this atom is that *x* and *y* are independent of each other in the sense that nothing can be said about the value of *x* based on the value of *y*, and vice versa. *Independence logic* even allows for atoms $\overline{x}\, \bot _{\overline{z}}\, \overline{y}$ which state that the tuples $\overline{x}$ and $\overline{y}$ are independent when the values of the variables in $\overline{z}$ are kept constant; see Grädel and Väänänen (2013) for the formal details.

Galliani (2012) introduced *inclusion logic*. This is yet a further variant of dependence logic. Inclusion logic extends first-order logic by atoms of type $\overline{x}\subseteq \overline{y}$ which state that any tuple of values defined by $\overline{x}$ is also a tuple of values defined by $\overline{y}$. Galliani (2012) also defined two separate systems of team semantics called *strict* and *lax* semantics. The systems differ from each other in their treatment of the existential quantifier and disjunction.

In strict semantics, the existential quantifier is treated in the original way familiar from dependence logic: a model $\mathfrak {A}$ and a team *X* satisfy a formula $\exists x\, \varphi$ if and only if it is possible to extend^2^ each valuation $s\in X$ with a pair $(x,a)$, where $a\in Dom(\mathfrak {A})$, such that the resulting extended team satisfies $\varphi$. The key issue here is that each valuation $s\in X$ is extended by *exactly one* pair $(x,a)$ that provides an interpretation for *x*. In lax semantics, each assignment $s\in X$ can be extended by more than one pair $(x,a)$, and this results in a whole set of extensions of the valuation *s*. For the technical difference between the strict and lax semantics in their treatment of the disjunction, see Galliani (2012) or Sect. 3 below.

There are interesting and perhaps surprizing differences between the lax and strict semantics. It has been shown by Galliani and Hella (2013) that with lax semantics, inclusion logic is equi-expressive with *positive greatest fixed point logic*, and therefore captures PTIME in restriction to linearly ordered finite models. On the other hand, it was observed by Galliani et al. (2013) that with strict semantics, inclusion logic captures NP.

In addition to extensions of first-order logic with different kinds of atoms, also *generalized quantifiers* have been studied in the context of team semantics. Engström (2012) defined a semantics that can accommodate generalized quantifiers in the framework of team semantics. Inter alia, Engström (2012) studied *branching quantifiers* consisting of partially ordered generalized quantifiers. Investigations in the setting of Engström (2012) have been recently continued for example in the articles Engström and Kontinen (2013) and Engström et al. (2013).

In this article, we define a novel semantics that can deal with extensions of dependence logic and its variants with generalized quantifiers. Our approach is based on *double teams* and differs significantly from the semantics in Engström (2012). There are several reasons—discussed below—why we believe that the *double team semantics* is particularly *natural, general, and useful*.

The semantics we shall define is *fully symmetric* in the sense that it respects obvious *canonical duality principles* concerning negation. The semantics is also *compositional for negation* in a very natural way. In investigations related to team semantics, the syntax of the logic studied is usually given in *negation normal form*, which means that negations are only allowed in front of atomic formulae.^3^ In the framework of double team semantics, such syntactic limitations are avoided.

In addition to the double team semantics, we also define a corresponding *canonical game-theoretic semantics* and prove equivalence of the two semantic frameworks. Importantly, the game-theoretic semantics gives a natural action-based framework that can be used in order to *interpret and analyze* the meaning of logical operators—such as quantifiers and connectives—whose semantics has been given using the double team semantics.

The double team semantics and its game theoretic counterpart provide a suitable setting for the definition of a novel notion of a *minor quantifier*. The definition is a slight generalization of Lindström’s definition of the notion of a generalized quantifier in Lindström (1966). The notion of a minor quantifier provides a *natural* way of accommodating the lax and strict versions of the existential quantifier under the same umbrella framework.

While the strict and lax existential quantifiers canonically give rise to two corresponding minor quantifiers, it turns out that the ordinary existential quantifier—viewed as a generalized quantifier—gives rise to a third minor quantifier different from the strict and lax operators. The semantic framework based on double teams provides a natural setting for *interpretation of the meaning* of the strict and lax quantifiers. In particular, the framework enables investigation of the relationship between ordinary generalized quantifiers and the strict and lax quantifiers, thereby providing novel *insight* into the nature of these formal tools that occupy an important role in current research on team semantics.

In addition to the notion of a minor quantifier, we introduce the notion of a *generalized atom*. Generalized atoms declare properties of (double) teams. The atoms $=\!\!(x_1,\ldots ,x_k)$, $\overline{x}\, \bot _{\overline{z}}\, \overline{y}$ and $\overline{x}\, \subseteq \, \overline{y}$ are examples of generalized atoms. In addition to minor quantifiers, the double team semantics and its game-theoretic counterpart accommodate generalized atoms under the same general system of semantics. Generalized atoms were originally defined in the technical report Kuusisto (2012).

Recent research on team semantics has revealed—as one perhaps could expect—that subtle changes in semantic choices, such as using the lax semantics instead of strict, can give rise to logics with different expressivities. To understand related phenomena better, it definitely makes sense to study systems based on team semantics in a general unified umbrella framework. The double team semantics provides such a framework.

In order to demonstrate how the double team semantics works in practice, we define a logic $\hbox {DC}^2$ which extends *two-variable dependence logic*$\hbox {D}^2$ by counting quantifiers $\exists ^{\ge k}$. We prove that the satisfiability problem of this logic is decidable. In fact, we show that both the finite and standard satisfiability problems of $\hbox {DC}^2$ are $\hbox {NEXPTIME}$-complete.

The logic $\hbox {DC}^2$ is an extension of both two-variable dependence logic $\hbox {D}^2$ and *two-variable logic with counting*$\hbox {FOC}^2$. It was shown by Pratt-Hartmann (2005) that the satisfiability and finite satisfiability problems of $\hbox {FOC}^2$ are $\hbox {NEXPTIME}$-complete. In Kontinen et al. (2011), the corresponding problems for $\hbox {D}^2$ were shown to also be $\hbox {NEXPTIME}$-complete.

Research on two-variable logics is currently particularly active. Recent articles in the field include for example Benaim et al. (2013), Charatonik and Witkowski (2013), Kieroński and Michaliszyn (2012), Kieroński et al. (2012), Manuel and Zeume (2013), Szwast and Tendera (2013), and several others. Mainly the related research has concerned decidability and complexity issues in restriction to particlar classes of structures, and also questions related to different built-in features and operators that increase the expressivity of the base language. Team semantics has so far been discussed in this context only in Kontinen et al. (2011).

The article Kontinen et al. (2011) discusses ordinary two-variable dependence logic $\hbox {D}^2$ which does not include counting quantifiers. In fact, when writing Kontinen et al. (2011), no direct semantics for counting quantifiers was available in the team semantics framework. The double team approach provides an appropriate canonical system of semantics, and furthermore, facilitates the $\hbox {NEXPTIME}$-completeness proof given below. Concerning the proof, our objective is not so much to study the particular logic $\hbox {DC}^2$. Instead, we wish to demonstrate how the double team framework can in practice be used in order to study fragments of team-semantics-based logics extended with generalized quantifiers.

The double team semantics provides a general system that can deal with generalized quantifiers as well as generalized atoms, but on the face of it, the move from single teams to double teams may seem like an undesirable step towards a more complicated framework. We claim that this issue is not so simple, for two reasons.

Firstly, the syntax of a typical currently studied variant of dependence logic gives formulae in negation normal form, leading to systems with more connectives and quantifiers than necessary. Disjunction and conjunction have to be *both* included as primitive connectives in the logics, and the same applies to the existential and universal quantifiers. In proofs, all connectives and quantifiers must be considered. In the framework of double team semantics, the number of necessary connectives is smaller, but the treatment of each operator can of course be more laborious. In any case, the double team semantics offers a more liberal syntax.

Secondly, as we shall demonstrate below in Sect. 10, it is not difficult to define a natural semantics which is rather similar to our double team semantics—facilitating investigations analogous to those carried out in this article—but formulated in terms of single teams.

Finally, it is worth noting that while the double team semantics can be used in investigations related to dependence logic and its variants, it is also a *canonical semantics for ordinary extensions of first-order logic with generalized quantifiers*, i.e., extensions that do not include generalized atoms.

The structure of this article is as follows. In Sects. 2 and 3, we discuss the necessary background definitions. In Sect. 4, we define the double team semantics and discuss some of its basic properties. In Sects. 5 and 6, we introduce and investigate generalized atoms and minor quantifiers. In Sect. 7, we define a game-theoretic counterpart for the double team semantics. We also show that the two systems of semantics are equivalent. In Sect. 8, we discuss issues related to the interpretation of quantifiers and atoms in the framework of team semantics. In Sect. 9, we investigate the logic $\hbox {DC}^2$. In particular, we prove $\hbox {NEXPTIME}$-completeness of the satisfiability and finite satisfiability problems of the logic. In Sect. 10, we briefly discuss a single team semantics for generalized quantifiers.

## Preliminaries

Let $\mathbb {Z}_+$ denote the set of positive integers, and let $\hbox {VAR} = \{\, v_i\ |\ i\in \mathbb {Z}_+\ \}$ be the set of exactly all first-order *variable symbols*. We shall mainly use metavariables $x,y,z,x_1,x_2$, etc., in order to refer to variable symbols in $\hbox {VAR}$. We let $\overline{x},\overline{y},\overline{z},\overline{x}_1,\overline{x}_2$, etc., denote finite nonempty tuples of variable symbols, i.e., tuples in $\hbox {VAR}^n$ for some $n\in \mathbb {Z}_+$.

Let $X\subseteq \hbox {VAR}$ be a *finite*, possibly empty set. Let $\mathfrak {A}$ be a model with domain *A*. We do not allow for models to have an empty domain, so $A\not =\emptyset$. A function $f:X\rightarrow A$ is called an *assignment* for the model $\mathfrak {A}$.

Let $\overline{a}$ be any finite nonempty tuple. We let $\overline{a}(k)$ denote the *k*-th member of the tuple: for example $(a,b)(1) = a$ and $(a,b)(2) = b$. When we write $u\in \overline{a}$, we mean that *u* is a member of the tuple $\overline{a}$, i.e., if $\overline{a} = (a_1,\ldots ,a_n)$, then $u\in \overline{a}$ iff $u\in \{a_1,\ldots ,a_n\}$. If *f* is a function mapping into some set $S^k$ of tuples of length $k\in \mathbb {Z}_+$, then $f_i$ denotes the function with the same domain as *f* defined such that

$$\begin{aligned} f_i(x)= \bigl (f(x)\bigr )(i), \end{aligned}$$

i.e., $f_i$ is the *i-th coordinate function* of *f*.

Let *s* be an assignment with domain *X* and for the model $\mathfrak {A}$. Let $n\in \mathbb {Z}_+$. Let $\overline{x}\in \hbox {VAR}^n$ be a finite nonempty tuple of variables, and let $\overline{a}\in A^n$. Assume that if $\overline{x}$ repeats a variable, then $\overline{a}$ repeats the corresponding value, i.e., if $\overline{x}(i)= \overline{x}(j)$ for some $i,j\in \{1,\ldots ,n\}$, then $\overline{a}(i)= \overline{a}(j)$. We say that $\overline{a}$*respects*$\overline{x}$*-repetitions*. We let $s[\overline{x}/ \overline{a}]$ denote the variable assignment for $\mathfrak {A}$ with the domain $X\cup \{\ x\ |\ x\in \overline{x}\ \}$ defined as follows.

1. $$s[\overline{x}/ \overline{a}](y) = \overline{a}(k)$$ if $$y = \overline{x}(k)$$,
2. $$s[\overline{x}/ \overline{a}](y) = s(y)$$ if $$y\not \in \overline{x}$$.

Let $T\in \fancyscript{P}(A^n)$, where $\fancyscript{P}$ denotes the power set operator and $n\in \mathbb {Z}_+$. Assume that each tuple in *T* respects $\overline{x}$-repetitions. We define

$$\begin{aligned} s[\overline{x}/ T\, ] = \{\ s[\, \overline{x}/ \overline{a}\, ]| \overline{a}\in T\}. \end{aligned}$$

Note that $s[\, \overline{x}/\emptyset \, ] = \emptyset$. Let *S* be a set and $\overline{z}$ a tuple of variables of length $k\in \mathbb {Z}_+$. If $T\subseteq S^k$ is a relation such each $\overline{u}\in T$ respects $\overline{z}$-repetitions, then we say that the relation *Trespects*$\overline{z}$-*repetitions.*

Let $X\subseteq \hbox {VAR}$ be a finite, possibly empty set of first-order variable symbols. Let *U* be a set of assignments $f:X\rightarrow A$. Such a set *U* is a *team* with *domainX* and for the model $\mathfrak {A}$. The domain *A* of the model $\mathfrak {A}$ is a *codomain* of the team *U*. Note that the empty set is a team for $\mathfrak {A}$, as is the set $\{\emptyset \}$ containing only the empty assignment. The team $\emptyset$ does not have a unique domain; any finite subset of VAR is a domain of $\emptyset$. The domain of the team $\{\emptyset \}$ is $\emptyset$.

A pair of teams $(U,V)$ is a *double team* if *U* and *V* are teams with the same domain; the pairs $(U,\emptyset )$, $(\emptyset ,V)$ are double teams when *U* and *V* are teams.

Let *V* be a team with domain *X* and for the model $\mathfrak {A}$. Let $n\in \mathbb {Z}_+$, and let $S\subseteq A^n$. Let $f:V\rightarrow \fancyscript{P}(S)$ be a function. Let $\overline{x} = (x_1,\ldots ,x_n)$ be a tuple of variables. Assume that for each $s\in V$, the relation $f(s)$ respects $\overline{x}$-repetitions. Then we say that *f respects*$\overline{x}$-*repetitions*. We define

$$\begin{aligned} V[\, \overline{x}/f\, ]=\bigcup \limits _{s\, \in \, V}s[\, \overline{x}/f(s)\, ]. \end{aligned}$$

Note that it $V=\emptyset$, then we have $V[\overline{x}/ f] = \emptyset$. Let *B* denote the set

$$\begin{aligned} \{\ \overline{a}\in A^n\ |\ \overline{a}\,\hbox {respects}\, \overline{x}\hbox {-repetitions}\ \}. \end{aligned}$$

We let $f':V\rightarrow \fancyscript{P}(B)$ denote the function defined such that $f'(s) = B\setminus f(s)$ for all $s\in V$. Naturally

$$\begin{aligned} V[\, \overline{x}/f'\, ]=\bigcup \limits _{s\, \in \, V}s[\, \overline{x}/f'(s)\, ]. \end{aligned}$$

Let *V* be a team with domain *X* and for the model $\mathfrak {A}$. Let $k\in \mathbb {Z}_+$. Let $y_1,\ldots ,y_k$ be variable symbols. Assume that $\{y_1,\ldots ,y_k\} \subseteq X$. Define

$$\begin{aligned} \hbox {Rel}\bigl (V,\mathfrak {A},(y_1,\ldots ,y_k)\bigr )= \{\ \bigl (s(y_1),\ldots ,s(y_k)\bigr )\ |\ s\in V\ \}. \end{aligned}$$

If *V* is empty, then the obtained relation is the empty relation. Notice that the relation $\hbox {Rel}\bigl (V,\mathfrak {A},(y_1,\ldots ,y_k)\bigr )$ does not depend on $\mathfrak {A}$, so we occasionally simply write $\hbox {Rel}\bigl (V,(y_1,\ldots ,y_k)\bigr )$ instead of $\hbox {Rel}\bigl (V,\mathfrak {A},(y_1,\ldots ,y_k)\bigr )$.

Let $(i_1,\ldots ,i_n)$ be a non-empty sequence of positive integers. A *generalized quantifier* (cf. Lindström 1966) of type $(i_1,\ldots ,i_n)$ is a class $\fancyscript{C}$ of structures $(A,B_1,\ldots ,B_n)$ such that the following conditions hold.

1. $$A\not =\emptyset $$.
2. For each $j\in \{1,\ldots ,n\}$, we have $B_j \subseteq A^{i_j}$.
3. If $(A',B_1',\ldots ,B_n')\in \fancyscript{C}$ and if there is an isomorphism $f:A'\rightarrow A''$ from $(A',B_1',\ldots ,B_n')$ to another structure $(A'',B_1'',\ldots ,B_n'')$, then we have $(A'',B_1'',\ldots ,B_n'')\in \fancyscript{C}$.

Let *Q* be a generalized quantifier of type $(i_1,\ldots ,i_n)$. We let $\overline{Q}$ denote the generalized quantifier of type $(i_1,\ldots ,i_n)$ defined such that

$$\begin{aligned} \overline{Q}=\{\ (A,C_1,\ldots ,C_n)\ |\ (A,C_1,\ldots ,C_n)\not \in Q\ \}. \end{aligned}$$

Let $\mathfrak {A}$ be a model with domain *A*. We define $Q^{\mathfrak {A}}$ to be the set

$$\begin{aligned} \{\ (B_1,\ldots ,B_n)\ |\ (A,B_1,\ldots ,B_n)\in Q\ \}. \end{aligned}$$

Similarly, we define

$$\begin{aligned} \overline{Q}^{\mathfrak {A}}= \{(B_1,\ldots ,B_n)\ |\ (A,B_1,\ldots ,B_n) \in \overline{Q}\}. \end{aligned}$$

If $\varphi$ is a formula of first-order logic, possibly extended with generalized quantifiers, we write $\mathfrak {A},s\models _{\mathrm{FO}}\varphi$ when the model $\mathfrak {A}$ satisfies $\varphi$ under the assignment *s*. The related semantic clause for generalized quantifiers is as follows.

Let *Q* denote a generalized quantifier of type $(i_1,\ldots ,i_n)$. Consider expressions of type $Q\overline{x}_1,\ldots ,\overline{x}_n(\varphi _1,\ldots ,\varphi _n)$, where $\overline{x}_j$ is a tuple of variables of length $i_j$ and $\varphi _j$ is a formula of first-order logic, possibly extended with generalized quantifiers. Let $\mathfrak {A}$ be a model with domain *A* and *s* an assignment with codomain *A*. If $\overline{x}$ is a tuple of variables of length $k\in \mathbb {Z}_+$, we let $A^k[\overline{x}]$ denote the set of exactly all tuples in $A^k$ that respect $\overline{x}$-repetitions. We define $\mathfrak {A},s\models _{\mathrm{FO}} Q\overline{x}_1,\ldots ,\overline{x}_n(\varphi _1,\ldots ,\varphi _n)$ iff $\bigl (\, A, S_1,\ldots ,S_n\, \bigr )\, \in \, Q^{\mathfrak {A}},$ where $S_j= \{\ \overline{a}\in A^{i_j}[\overline{x}_j]\ |\ \mathfrak {A},s[\overline{x}_j/ \overline{a}]\models _{\mathrm{FO}}\varphi _j\ \}.$ The quantifier $Q\overline{x}_1,\ldots ,\overline{x}_n$ binds the variables $\overline{x}_j$ in the formula $\varphi _j$. We of course assume that *s* interprets all the free variables in the formula $Q\overline{x}_1,\ldots ,\overline{x}_n(\varphi _1,\ldots ,\varphi _n)$ and that $\mathfrak {A}$ interprets the non-logical symbols that appear in the formulae $\varphi _1,\ldots ,\varphi _n$.

Below, once we have defined the notion of a minor quantifier, we occasionally call generalized quantifiers *ordinary generalized quantifiers*.

In the investigations below, each instance of a subformula of a formula $\varphi$ is considered to be a distinct subformula: for example, in the formula $(P(x)\vee P(x))$, the left and right instances of the formula $P(x)$ are considered to be two *distinct* subformulae of the formula $(P(x)\vee P(x))$. It is not important how this distinction is achieved formally.

We let $\hbox {SUB}_{\varphi }$ denote the set of subformulae of the formula $\varphi$. For example, the set $\hbox {SUB}_{(P(x)\vee P(x))}$ has three subformulae in it, the formula $(P(x)\vee P(x))$ and both instances of $P(x)$.

We consider only models with a purely relational vocabulary, without function symbols or constant symbols. When we informally leave brackets unwritten in formulae, the order of priority of binary connectives is such that $\wedge$ is first, and then come $\vee$, $\rightarrow$ and $\leftrightarrow$, in the given order. The notation $\mathfrak {A},[x\mapsto a,y\mapsto b]\models _{\mathrm{FO}}\varphi$ means that we have $\mathfrak {A},s\models _{\mathrm{FO}}\varphi$, where *s* is an assignment whose domain is $\{x,y\}$, and it holds that $s(x) = a$ and $s(y) = b$. If *U* is a team, we let $Dom(U)$ denote the domain of the team. Similarly, if $\mathfrak {A}$ is a model, we let ${Dom}(\mathfrak {A})$ denote the domain of $\mathfrak {A}$.

## Dependence Logic and its Variants

Let $\tau$ be a vocabulary containing relation symbols only. Let $\fancyscript{A}(\tau )$ be the smallest set *T* such that the following conditions are satisfied.

1. Let $x_1$ and $x_2$ be (not necessarily distinct) variable symbols. Then $x_1 = x_2\ \in \ T$.
2. Let *k* be a positive integer. If $R\in \tau$ is a *k*-ary relation symbol and $x_1,\ldots ,x_k$ are (not necessarily distinct) variable symbols, then $R(x_1,\ldots ,x_k)\in T$.
3. Let *k* be a positive integer. If $x_1,\ldots ,x_k$ are (not necessarily distinct) variable symbols, then $=\!\!(x_1,\ldots ,x_k)\in T$.

Formulae formed by the rules 1 and 2 above are called *first-order atoms*. The set of $\tau$-formulae of *dependence logic*$\hbox {D}$ is the smallest set *T* such that the following conditions hold.

1. $$\fancyscript{A}(\tau )\subseteq T$$.
2. If $$\varphi \in \fancyscript{A}(\tau )$$, then $$\lnot \varphi \in T$$.
3. If $$\varphi ,\psi \in T$$, then $$(\varphi \vee \psi )\in T$$.
4. If $$\varphi ,\psi \in T$$, then $$(\varphi \wedge \psi )\in T$$.
5. If $\varphi \in T$ and $z\in \hbox {VAR}$, then $\exists z\, \varphi \in T$.
6. If $\varphi \in T$ and $z\in \hbox {VAR}$, then $\forall z\, \varphi \in T$.

*Two-variable dependence logic*$\hbox {D}^2$ is a fragment of *D*. Let $\sigma$ be a vocabulary containing relation symbols only, and assume that each symbol in $\sigma$ is either of arity *1* or *2*. Fix two distinct variable symbols *x* and *y*. The set $\fancyscript{A}(\sigma )$ of atomic $\sigma$-formulae of $\hbox {D}^2$ is the smallest set *T* defined as follows.

1. Assume $P\in \sigma$ and $R\in \sigma$ are unary and binary relation symbols, respectively. Let $z,z'\in \{x,y\}$ be (not necessarily distinct) variables. Then $P(z)\in T$ and $R(z,z')\in T$.
2. Let $z,z'\in \{x,y\}$ be (not necessarily distinct) variables. Then we have $=\!\!(z)\in T$ and $=\!\!(z,z')\in T$. Also $z = z'\in T$.

The set of $\sigma$-formulae of $\hbox {D}^2$ is the smallest set *T* satisfying the following conditions.

1. $$\fancyscript{A}(\sigma )\subseteq T$$.
2. If $$\varphi \in \fancyscript{A}(\sigma )$$, then $$\lnot \varphi \in T$$.
3. If $$\varphi ,\psi \in T$$, then $$(\varphi \vee \psi )\in T$$.
4. If $$\varphi ,\psi \in T$$, then $$(\varphi \wedge \psi )\in T$$.
5. If $\varphi \in T$ and $z\in \{x,y\}$, then $\exists z\, \varphi \in T$.
6. If $\varphi \in T$ and $z\in \{x,y\}$, then $\forall z\, \varphi \in T$.

We next define the semantics of $\hbox {D}$. In the definition, $\mathfrak {A}$ denotes a model and *U* a team. The domain of the team *U* is always assumed to contain the free variables in the formulae, and the codomain of *U* is of course assumed to be the domain *A* of the model $\mathfrak {A}$. Furthermore, it is assumed that the vocabulary of the model $\mathfrak {A}$ contains the non-logical symbols in the formulae. The following clauses define the semantics of dependence logic D.

$$\begin{aligned} \begin{array}{lll} \mathfrak {A}\models _U x_1=x_2 &{} \ \Leftrightarrow \ &{} \forall s\in U\bigl (\mathfrak {A},s\models _{\mathrm{FO}} x_1=x_2\bigr ).\\ \mathfrak {A}\models _U R(x_1,\ldots ,x_m)&{} \ \Leftrightarrow \ &{} \forall s\in U\bigl (\mathfrak {A},s\models _{\mathrm{FO}} R(x_1,\ldots ,x_m)\bigr ).\\ \mathfrak {A}\models _U\ =\!\!(x_1,\ldots ,x_m)&{} \ \Leftrightarrow \ &{} \hbox {if there exist assignments}\,s,t\in U\hbox { such that}\\ &{}&{}s(x_i) = t(x_i)\,\hbox {for all}\,i\in \{1,\ldots ,m\}\setminus \{m\},\\ &{}&{}\hbox {then we have}\,s(x_m) = t(x_m).\\ \mathfrak {A}\models _U \lnot \, x_1=x_2 &{} \ \Leftrightarrow \ &{} \forall s\in U\bigl (\mathfrak {A},s\not \models _{\mathrm{FO}} x_1=x_2\bigr ).\\ \mathfrak {A}\models _U \lnot \, R(x_1,\ldots ,x_m)&{} \ \Leftrightarrow \ &{} \forall s\in U\bigl (\mathfrak {A},s\not \models _{\mathrm{FO}} R(x_1,\ldots ,x_m)\bigr ).\\ \mathfrak {A}\models _U \lnot =\!\!(y_1,\ldots ,y_m)&{} \ \Leftrightarrow \ &{} U = \emptyset .\\ \mathfrak {A}\models _U (\varphi \vee \psi ) &{} \ \Leftrightarrow \ &{} \mathfrak {A}\models _{U_1}\varphi \,\hbox {and} \mathfrak {A}\models _{U_2}\psi \,\hbox {for some}\\ &{} &{}U_1,U_2\subseteq U\,\hbox {such that}\,U_1\cup U_2 = U.\\ \mathfrak {A}\models _U (\varphi \wedge \psi ) &{} \ \Leftrightarrow \ &{} \mathfrak {A}\models _{U_1}\varphi \,\hbox {and} \mathfrak {A}\models _{U_2}\psi .\\ \mathfrak {A}\models _U \exists z\, \varphi &{} \ \Leftrightarrow \ &{} \mathfrak {A}\models _{U[z/f]}\varphi \,\hbox {for some}\,f:U\rightarrow \{\ \{a\}\ |\ a\in A\ \}.\\ \mathfrak {A}\models _U \forall z\, \varphi &{} \ \Leftrightarrow \ &{} \mathfrak {A}\models _{U[z/A]}\varphi . \end{array} \end{aligned}$$

Notice that $\mathfrak {A}\models _{U}\, =\!\!(z)$ iff $s(z) = s'(z)$ for all $s,s'\in U$. If $\varphi$ is a sentence, then we define $\mathfrak {A}\models \varphi$ iff $\mathfrak {A}\models _{\{\emptyset \}}\varphi$.

To get a taste of how dependence logic works, consider the formula

$$\begin{aligned} \forall x \forall y\bigl ( \lnot R(x,y) \vee (R(x,y)\, \wedge \, =\!\!(x,y))\bigr ). \end{aligned}$$

As the reader can verify, this formula states that the out-degree of *R* is at most one everywhere. For another example, consider the formula

$$\begin{aligned} \forall x\exists y\bigl ( =\!\!(x,y)\, \wedge \, R(x,y) \bigr ). \end{aligned}$$

This formula states that the relation *R* is a function.

Formulae of $\hbox {D}$ that do not contain instances of atoms $=\!\!(x_1,\ldots ,x_k)$ are called *first-order formulae*. It is well-known and easy to show that for first-order formulae, $\mathfrak {A}\models _{U}\varphi$ iff we have $\mathfrak {A},s\models _{\mathrm{FO}}\varphi$ for all $s\in U$.

Variants of dependence logic studied in the current literature include for example *inclusion logic* Galliani (2012). The syntax of inclusion logic is the same as that of dependence logic, with the exception that instead  of atomic expressions $=\!\!(x_1,\ldots ,x_m)$, the non-first-order atoms in inclusion logic are *inclusion atoms*

$$\begin{aligned} (y_1,\ldots ,y_k)\, \subseteq \, (z_1,\ldots ,z_k), \end{aligned}$$

and negated inclusion atoms are not allowed at all. Inclusion atoms are interpreted such that $\mathfrak {A}\models _{U} (y_1,\ldots ,y_k)\, \subseteq \, (z_1,\ldots ,z_k)$ if and only if

$$\begin{aligned} {Rel}\bigl (U,\mathfrak {A},(y_1,\ldots ,y_k)\bigr ) \subseteq {Rel}\bigr (U,\mathfrak {A},(z_1,\ldots ,z_k)\bigr ). \end{aligned}$$

The existential quantifier is interpreted such that $\mathfrak {A}\models _{U}\exists z\, \varphi$ iff $\mathfrak {A}\models _{U[z/f]}\varphi$ for some function $f:U\rightarrow \bigl (\fancyscript{P}(A)\setminus \emptyset \bigr )$. Other semantic clauses are exactly the same as the ones given for dependence logic above. This results in the interpretation of inclusion logic with *lax semantics*.

Inclusion logic can also be interpreted using *strict semantics*. The difference between strict and lax semantics concerns the interpretation of the existential quantifier and disjunction. For the existential quantifier, the semantic clause in strict semantics is exactly the same as the clause given for dependence logic above. For the disjunction, the semantic clause in strict semantics dictates that $\mathfrak {A}\models _{U}\varphi \vee \psi$ iff we have $\mathfrak {A}\models _{U_1} \varphi$ and $\mathfrak {A}\models _{U_2}\psi$ for some teams $U_1,U_2\subseteq U$ such that $U_1\cup U_2 = U$ and $U_1\cap U_2 = \emptyset$.

Galliani and Hella (2013) have established that with lax semantics, inclusion logic is equi-expressive with the *positive greatest fixed point logic* and therefore captures PTIME in restriction to ordered finite models. With strict semantics, inclusion logic captures NP, as observed by Galliani et al. (2013).

Also *independence logic*, defined by Grädel and Väänänen (2013), is a widely studied variant of dependence logic. For formal details related to independence logic, see Grädel and Väänänen (2013).

Logics in the family of dependence logic and independence-friendly logic have unorthodox properties that may appear strange. For example, it is easy to see that if $\mathfrak {A}$ is a model with exactly two elements, then under strict semantics, we have $\mathfrak {A}\not \models _{\{\emptyset \}} \exists x \forall y\, y\subseteq x$, while $\mathfrak {A}\models _{\{\emptyset \}}\forall z \exists x \forall y\, y\subseteq x$. We also have $\mathfrak {A}\not \models _{\{\emptyset \}}\forall x\, =\!\!(x)$, while $\mathfrak {A}\models _{\{\emptyset \}}\forall x\bigr (=\!\!(x)\, \vee \, =\!\!(x)\, \bigr )$.

## A Double Team Semantics

In ordinary team semantics, the *background intuition*^4^ concerning satisfaction of formulae is that a team satisfies a formula $\varphi$ iff every member of the team satisfies $\varphi$. In the double team semantics we shall define below, the background intuition is that a double team (*U*,*V*) satisfies a formula iff every assignment in the team *U* satisfies the formula, and furthermore, every assignment in the team *Vfalsifies* the formula. Both in ordinary and double team semantics, the intuition is actually even formally valid when the investigated formula is a first-order formula.

The truth definition for first-order atoms and connectives is as follows.

$$\begin{aligned} \begin{array}{lll} \mathfrak {A},(U,V)\models x_1=x_2 &{} \ \Leftrightarrow \ &{} \forall s\in U\bigl (\mathfrak {A},s\models _{\mathrm{FO}} x_1=x_2\bigr )\hbox { and }\\ &{} &{} \forall s\in V\bigl (\mathfrak {A},s\not \models _{\mathrm{FO}} x_1=x_2\bigr ).\\ \mathfrak {A},(U,V)\models R(x_1,\ldots ,x_m)&{} \ \Leftrightarrow \ &{} \forall s\in U\bigl (\mathfrak {A},s\models _{\mathrm{FO}} R(x_1,\ldots ,x_m)\bigr )\hbox { and }\\ &{} &{} \forall s\in V\bigl (\mathfrak {A},s\not \models _{\mathrm{FO}} R(x_1,\ldots ,x_m)\bigr ).\\ \mathfrak {A},(U,V)\models \lnot \varphi &{} \ \Leftrightarrow \ &{} \mathfrak {A},(V,U)\models \varphi .\\ \mathfrak {A},(U,V)\models (\varphi \vee \psi ) &{} \ \Leftrightarrow \ &{} \mathfrak {A},(U_1,V)\models \varphi \hbox { and } \mathfrak {A},(U_2,V)\models \psi \hbox { for}\\ &{} &{}\hbox {some }U_1,U_2\subseteq U\hbox { such that } U_1\cup U_2 = U. \end{array} \end{aligned}$$

Here we of course assume that the domain of *U* and *V* contains the free variables in the formulae, and that $\mathfrak {A}$ interprets the non-logical symbols that occur in the formulae.

We next extend our framework of double team semantics to generalized quantifiers. The definition is given for quantifiers of any type $(i_1,\ldots ,i_n)$, but it may be instructive to consider the details of the definition in the special case of quantifiers of type $(1)$. For the sake of simplicity, our investigations below concern mostly only quantifiers of type $(1)$.

The background intuition concerning the satisfaction of  a  quantified  formula  $Qx\, \varphi (x)$ is based on the idea that the set of witnesses of $Qx\, \varphi (x)$ is the set of *exactly all* values *b* such that $\varphi (b)$ holds. A proper subset will not do. This intuition easily generalizes to generalized quantifiers *Q* of arbitrary types. For a generalized quantifier *Q* of type $(i_1,\ldots ,i_n)$, we define

$$\begin{aligned} \mathfrak {A},(U,V)\models Q\overline{x}_1,\ldots ,\overline{x}_n(\varphi _1,\ldots ,\varphi _n) \end{aligned}$$

if and only if there exist functions $f:U\rightarrow Q^{\mathfrak {A}}$ and $g:V\rightarrow \overline{Q}^{\mathfrak {A}}$ such that

$$\begin{aligned} \begin{array}{c} \mathfrak {A},\bigl (\, U[\, \overline{x}_1/f_1]\cup V[\, \overline{x}_1/{g_1}\, ], \, U[\, \overline{x}_1/{f_1}'\, ]\cup V[\, \overline{x}_1/{g_1}'\, ]\, \bigr )\models \varphi _1,\\ \vdots \\ \mathfrak {A},\bigl (\, U[\, \overline{x}_n/f_n]\cup V[\, \overline{x}_n/{g_n}\, ], \, U[\, \overline{x}_n/{f_n}'\, ]\cup V[\, \overline{x}_n/{g_n}'\, ]\, \bigr )\models \varphi _n.\\ \end{array} \end{aligned}$$

The functions *f* and *g* must have the property that for each $i\in \{1,\ldots ,n\}$, the coordinate functions $f_i$ and $g_i$ (and also the functions $f_i'$ and $g_i'$) respect $\overline{x}_i$-repetitions.

Notice that if $W = \emptyset$ is the empty team and $f:W\rightarrow Q^{\mathfrak {A}}$ the empty function ($f = \emptyset$), then $W[\overline{x}/f] = \emptyset$.

### **Proposition 1**

Let $\varphi$ be a formula of first-order logic, possibly extended with generalized quantifiers. Let $(U,V)$ be a double team. Then

$$\begin{aligned} \mathfrak {A},(U,V)\models \varphi \ \hbox { iff }\ \forall s\in U\forall t\in V\bigl (\mathfrak {A},s\models _{\mathrm{FO}} \varphi \,\hbox {and}\,\mathfrak {A},t\not \models _{\mathrm{FO}}\varphi \bigr ). \end{aligned}$$

### *Proof*

The claim is established by a straightforward induction on the structure of formulae. $\square$

When $\varphi$ is a sentence, we define $\mathfrak {A}\models \varphi$ iff $\mathfrak {A},(\{\emptyset \},\emptyset )\models \varphi$. When $\mathfrak {A}$ is known from the context, we may write $(U,V)\models \psi$ instead of $\mathfrak {A},(U,V)\models \psi$.

Note that the truth definition of disjunction could be easily modified without sacrificing Proposition 1. For example we could define $\mathfrak {A},(U,V)\models \varphi \vee \psi$ iff $\mathfrak {A},(U_1,V\cup \, U_1')\models \varphi$ and $\mathfrak {A},(U_2,V\cup \, U_2')\models \psi$ for some $U_1,U_2\subseteq U$ such that $U_1\cup U_2 = U$; here $U_1' = U\setminus U_1$ and $U_2' = U\setminus U_2$. This definition would perhaps be a better match for our truth definition concerning generalized quantifiers. For the sake of simplicity, we shall mostly ignore such alternative definitions for connectives in this article. However, let us define the connective $\vee ^s$ such that $\mathfrak {A},(U,V)\models \varphi \vee ^s\psi$ iff $\mathfrak {A},(U_1,V)\models \varphi$ and $\mathfrak {A},(U_2,V)\models \psi$ for some $U_1,U_2\subseteq U$ such that $U_1\cup U_2 = U$ and $U_1\cap U_2 = \emptyset$.

## Generalized Atoms

Let *m* and *n* be non-negative integers such that $n+m > 0$. Let *Q* be a generalized quantifier of type $(i_1,\ldots ,i_{n+m})$. Consider atomic expressions of type

$$\begin{aligned} A_{Q,n}(\overline{y}_1,\ldots ,\overline{y}_n\, ;\, \overline{y}_{n+1},\ldots ,\overline{y}_{n+m}), \end{aligned}$$

where each $\overline{y}_j$ is a tuple of variables of length $i_j$, and $A_{Q,n}$ is simply a symbol. Extend the double team semantics such that

$$\begin{aligned} \mathfrak {A},(U,V)\models A_{Q,n}(\overline{y}_1,\ldots , \overline{y}_n\, ; \, \overline{y}_{n+1},\ldots ,\overline{y}_{n+m}) \end{aligned}$$

if and only if

$$\begin{aligned} \bigl (\hbox {Rel}(U,\mathfrak {A},\overline{y}_1),\ldots , \hbox {Rel}(U,\mathfrak {A},\overline{y}_n),\hbox {Rel}(V,\mathfrak {A},\overline{y}_{n+1}),\ldots , \hbox {Rel}(V,\mathfrak {A},\overline{y}_{n+m})\bigr )\in Q^{\mathfrak {A}}. \end{aligned}$$

The generalized quantifier *Q* and the number *n* define a *generalized atom* of type

$$\begin{aligned} \bigl ((i_1,\ldots ,i_n),(i_{n+1},\ldots ,i_{n+m})\bigr ). \end{aligned}$$

Note that types of generalized quantifiers are tuples and types of generalized atoms are pairs of tuples; exactly one tuple of such a pair of tuples can be the empty tuple. (We shall consider neither generalized atoms of type $(\emptyset ;\emptyset )$ nor generalized quantifiers of type $\emptyset$.)

We occasionally call generalized atoms *non-first-order atoms*. Other atoms are called first-order atoms.

## Minor Quantifiers

In this section we generalize the notion of a generalized quantifier given by Lindström (1966). We call the novel operators *minor quantifiers*. Minor quantifiers have a natural intuitive game-theoretic interpretation. In addition to the current section, issues concerning the interpretation will be discussed in Sects. 7 and 8. *The intuition behind minor quantifiers is best illuminated by the game-theoretic semantics given in Section* 7; *it will quite likely be helpful to keep this in mind while going through the technical definitions in the current section.*

Let *Q* be a generalized quantifier of type $(1)$. Let $\fancyscript{C}$ be a class of structures $(A,B_+,B_-)$ such that the following conditions hold.

1. $$A\not =\emptyset $$.
2. $$B_+\subseteq A$$ and $$B_-\subseteq A$$.
3. $$B_+\cap B_- = \emptyset $$.
4. If $(C,D_+,D_-)\in \fancyscript{C}$ and if there is an isomorphism $f:C\rightarrow E$ from $(C,D_+,D_-)$ to another structure $(E,F_+,F_-)$, then $(E,F_+,F_-)\in \fancyscript{C}$.
5. For each $(A,B_+,B_-)\in \fancyscript{C}$, there exists a pair $(A,H)\in Q$ such that $B_+\subseteq H$ and $B_-\subseteq A\setminus H$.
6. If $(A,B_+,B_-)\in \fancyscript{C}$, there does *not* exist a pair $(A,H)\in \overline{Q}$ such that $B_+\subseteq H$ and $B_-\subseteq A\setminus H$.
7. For each $(A,H)\in Q$, there exists a tuple $(A,B_-,B_-)\in \fancyscript{C}$ such that $B_+\subseteq H$ and $B_-\subseteq A\setminus H$.

We say that $\fancyscript{C}$*witnessesQ*.

Consider a pair $(\fancyscript{C},\fancyscript{D})$ such that $\fancyscript{C}$ witnesses *Q* and $\fancyscript{D}$ witnesses $\overline{Q}$. Here *Q* is a quantifier of type $(1)$. The pair $(\fancyscript{C},\fancyscript{D})$ defines a *minor quantifier* of type $(1)$. (For the sake of simplicity, we shall not define minor quantifiers of any other type.) Let $M = (\fancyscript{C},\fancyscript{D})$. We call *M* a *minor ofQ*. We write $M\le Q$.

A possible *intuitive* interpretation concerning the relationship between *Q* and a minor quantifier $M\le Q$ is that in order to verify $Qx\, \varphi (x)$ in a model $\mathfrak {A}$, one does not necessarily have to be able to find the set $B\in Q^{\mathfrak {A}}$ such that $b\in B$ iff $\varphi (b)$ holds in $\mathfrak {A}$. Depending on the quantifier *Q*, it may be enough to find some smaller set $B_+\subseteq B$ of values that verify $\varphi (x)$, possibly together with a set $B_-\subseteq {Dom}(\mathfrak {A})\setminus B$ of falsifying values. A tuple $(A,B_+,B_-)\in \fancyscript{C}$ then provides the sets $B_+$ and $B_-$. On the other hand, to *falsify* a formula $Qx\, \psi (x)$, it suffices to find a tuple $(A,E_+,E_-)$ in $\fancyscript{D}$, where $E_+$ is a set of verifying and $E_-$ a set of falsifying values for $\psi (x)$.

Therefore minor quantifiers provide a generalized perspective on generalized quantifiers. The perspective in some intuitive sense deals with issues concerning the *constructive verification and falsification* of formulae.

The semantics of minor quantifiers will be highly analogous to that of ordinary generalized quantifiers. To make this issue explicit, let us fix some notational conventions.

Let $M = (\fancyscript{C},\fancyscript{D})$ be a minor quantifier. Let $\mathfrak {A}$ be a model with domain *A*. Define

$$\begin{aligned} M^{\mathfrak {A}} = \{\ (B_+,B_-)\ |\ (A,B_+,B_-)\in \fancyscript{C}\ \} \end{aligned}$$

and

$$\begin{aligned} \overline{M}^{\mathfrak {A}} = \{\ (B_+,B_-)\ |\ (A,B_+,B_-)\in \fancyscript{D}\ \}. \end{aligned}$$

Let *U* be a team and $f:U\rightarrow M^{\mathfrak {A}}$ a function. *When discussing the semantics of minor quantifiers*$M = (\fancyscript{C},\fancyscript{D})$, *we mostly let*$U[ x/f ]$*denote the team*$U[ x/f_1 ]$, *while*$U[ x/f' ]$*denotes the team*$U[ x/f_2 ]$. Here $f_1$ and $f_2$ are the coordinate functions of *f*. Similarly, if *V* is a team and $g:V\rightarrow \overline{M}^{\mathfrak {A}}$ a function, we let $V[ x/g ]$ denote the team $U[ x/g_1 ]$ and $U[ x/g' ]$ the team $U[ x/g_2 ]$. This convention makes the connection between ordinary generalized quantifiers and minor quantifiers fully explicit. All related arguments will be carefully developed below, so no notational confusion arises. The only exception to this convention is the proof of Proposition 2.

Let *M* be a minor quantifier of type (1). Consider expressions of type $Mx\, \varphi$. Extend the double team semantics such that $\mathfrak {A},(U,V)\models Mx\, \varphi$ iff there exist functions $f:U\rightarrow M^{\mathfrak {A}}$ and $g:V\rightarrow \overline{M}^{\mathfrak {A}}$ such that

$$\begin{aligned} \mathfrak {A},\bigl (U[\, x/f\, ]\cup V[\, x/g\, ], \, U[\, x/f\, '\, ]\cup V[\, x/g\, '\, ]\bigr )\models \varphi . \end{aligned}$$

Notice that the form of the above semantic clause is now the same as in the case of ordinary quantifiers of type $(1)$.

The following proposition is easy to establish.

### **Proposition 2**

Let *Q* be a generalized quantifier and $M\le Q$ a minor quantifier. Let $\varphi$ be a formula of first-order logic extended with any collection of minor quantifiers and ordinary generalized quantifiers. Let $\varphi '$ be a formula obtained from $\varphi$ by replacing any occurrence of *Q* by *M*, or alternatively, any occurrence of *M* by *Q*. Then $\mathfrak {A},(U,V)\models \, \varphi \, \hbox {iff}\, \mathfrak {A},(U,V)\models \, \varphi '.$

### *Proof*

The claim of the proposition follows by Proposition by 1 from the properties of minor quantifiers. Let $\chi$ be a formula of first-order logic extended with any collection of minor quantifiers and ordinary generalized quantifiers. We will establish that $\mathfrak {A},(U,V)\models Mx\, \chi$ iff $\mathfrak {A},(U,V)\models Qx\, \chi$.

Assume that $\mathfrak {A},(U,V)\models Mx\, \chi$. Thus there exist functions $h:U\rightarrow M^{\mathfrak {A}}$ and $k:V\rightarrow \overline{M}^{\mathfrak {A}}$ such that

$$\begin{aligned} \mathfrak {A},\bigl (U[\, x/h_1\, ]\cup V[\, x/k_1\, ], \, U[\, x/h_2\, ]\cup V[\, x/k_2\, ]\bigr )\models \varphi . \end{aligned}$$

Let $s\in U$. Since $M\le Q$, there exist a pair $(A,H_s)\in Q$ such that $h_1(s)\subseteq H_s$ and $h_2(s)\subseteq A\setminus H_s$. Furthermore, there does not exist a pair $(A,H')\in \overline{Q}$ such that $h_1(s)\subseteq H'$ and $h_2(s)\subseteq A\setminus H'$. Therefore we conclude that $\mathfrak {A},t\models _{\mathrm{FO}}\varphi$ for each $t\in s[x / H_s]$ and $\mathfrak {A},r\not \models _{\mathrm{FO}}\varphi$ for each $r\in s[x / (A\setminus H_s) ]$. Define a function $f:U\rightarrow Q^{\mathfrak {A}}$ such that $f(s) = H_s$ for each $s\in H$.

Let $s\in V$. Since $M\le Q$, there exist a pair $(A,H_s)\in \overline{Q}$ such that $k_1(s)\subseteq H_s$ and $k_2(s)\subseteq A\setminus H_s$. Furthermore, there does not exist a pair $(A,H')\in Q$ such that $k_1(s)\subseteq H'$ and $k_2(s)\subseteq A\setminus H'$. Therefore we conclude that $\mathfrak {A},t\models _{\mathrm{FO}}\varphi$ for each $t\in s[x / H_s]$ and $\mathfrak {A},r\not \models _{\mathrm{FO}}\varphi$ for each $r\in s[x / (A\setminus H_s)]$. Define a function $g:U\rightarrow \overline{Q}^{\mathfrak {A}}$ such that $g(s) = H_s$ for each $s\in H$.

We have

$$\begin{aligned} \mathfrak {A},\bigl (U[\, x/f\, ]\cup V[\, x/g\, ], \, U[\, x/f\, '\, ]\cup V[\, x/g\, '\, ]\bigr )\models \varphi , \end{aligned}$$

whence $$\mathfrak {A},(U,V)\models Qx\varphi $$.

The converse implication is similar. Assume that $\mathfrak {A},(U,V)\models Qx\varphi$. Thus

$$\begin{aligned} \mathfrak {A},\bigl (U[\, x/f\, ]\cup V[\, x/g\, ], \, U[\, x/f\, '\, ]\cup V[\, x/g\, '\, ]\bigr )\models \varphi \end{aligned}$$

for some functions $f:U\rightarrow Q^{\mathfrak {A}}$ and $g:V\rightarrow \overline{Q}^{\mathfrak {A}}$.

Let $s\in U$. As $M\le Q$, there exists a pair $(B_+,B_-)_s\in M^{\mathfrak {A}}$ such that $B_+ \subseteq f(s)$ and $B_-\subseteq f'(s)$. Define a function $h:U\rightarrow M^{\mathfrak {A}}$ such that $h(s) = (B_+,B_-)_s$ for each $s\in U$. Analogously, define a function $k:V\rightarrow \overline{M}^{\mathfrak {A}}$ such that for each $r\in V$, we have $k(r) = (B_+,B_-)_r$, where $B_+\subseteq g(r)$ and $B_-\subseteq g'(r)$. We have

$$\begin{aligned} \mathfrak {A},\bigl (U[\, x/h_1\, ]\cup V[\, x/k_1\, ], \, U[\, x/h_2\, ]\cup V[\, x/k_2\, ]\bigr )\models \varphi , \end{aligned}$$

whence $$\mathfrak {A},(U,V)\models Mx\varphi $$.$$\square $$

Let *Q* be a generalized quantifier of type $(1)$. Notice that *Q* canonically defines the minor quantifier

$$\begin{aligned} M_Q\ :=\ \Bigl (\{\ (A,B,A\setminus B)\ |\ (A,B)\in Q\ \}, \{\ (A,B,A\setminus B)\ |\ (A,B)\in \overline{Q}\ \}\Bigr ) \end{aligned}$$

whose semantics is equivalent to that of *Q* in the double team framework: we can replace any instance of  *Q* by $M_Q$ (or vice versa) in any formula $\varphi$, and exactly the same models and double teams will satisfy the two formulae.^5^ We call $M_Q$*the minor quantifier defined by Q*. Ordinary generalized quantifiers can therefore be seen as special cases of minor quantifiers.

Define the *strict existential quantifier*$\exists ^s$ to be the minor quantifier $(\fancyscript{C},\fancyscript{D})$, where $\fancyscript{C}$ contains exactly all triples $(A,B,C)$ such that *A* is a nonempty set, $B\subseteq A$ is a singleton set, and $C = \emptyset$, while $\fancyscript{D}$ contains exactly all triples $(D,E,F)$ such that *D* is a nonempty set, $E = \emptyset$, and $F = D$. Define the *lax existential quantifier*$\exists ^l$ to be the minor quantifier $(\fancyscript{C},\fancyscript{D})$, where $\fancyscript{C}$ contains exactly all triples $(A,B,C)$ such that *A* is a nonempty set, $B\subseteq A$ is a nonempty set, and $C = \emptyset$, while $\fancyscript{D}$ contains exactly all triples $(D,E,F)$ such that *D* is a nonempty set, $E = \emptyset$, and $F = D$. Note that neither $\exists ^s$ nor $\exists ^l$ is equal to the minor quantifier $M_{\exists }$ defined by the ordinary existential quantifier.

## Game-Theoretic Semantics

In this section we define a natural game-theoretic semantics for first-order logic extended with all ordinary generalized quantifiers of type $(1)$, all minor quantifiers of type $(1)$, and all generalized atoms. We only deal with quantifiers of type $(1)$ in the rest of the article for the sake of simplicity. For game-theoretic approaches to the semantics of dependence logic and its variants, see for example Bradfield (2013), Galliani (2012), Grädel (2013), and Väänänen (2007). The semantics we shall define is based on the approach introduced in the technical report Kuusisto (2012).

Strictly speaking, we could of course avoid discussing ordinary generalized quantifiers here since they are essentially special cases of minor quantifiers, but we shall discuss them anyway since it makes the exposition of the background intuitions behind the game-theoretic semantics particularly transparent.

Let $\mathfrak {A}$ be a model with domain *A*. Let *s* be an assignment that maps a finite set of first-order variable symbols into *A*. We define a semantic game $G(\mathfrak {A},s,\#,\varphi )$, where $\#\in \{+,-\}$ is a symbol and $\varphi$ a formula. Here we assume that the assignment *s* interprets all the free variables in $\varphi$.

The game is played by an *agent*$\fancyscript{A}$ against an *interrogator*$\fancyscript{I}$. The intuition is that the interrogator poses questions, and the agent tries to answer them. In a game $G(\mathfrak {A},s,+,\varphi )$, the agent’s task is to maintain that $\varphi$ holds, while in a game $G(\mathfrak {A},s,-,\varphi )$, the agent’s task is to maintain that $\varphi$ does not hold.

A *play* of the game $G(\mathfrak {A},s,\#,\varphi )$ begins from the *position*$(\mathfrak {A},s,\#,\varphi )$. All positions of the game are tuples of the form $(\mathfrak {A},t,\#,\psi )$, where *t* is an assignment for $\mathfrak {A}$, $\#\in \{+,-\}$, and $\psi$ is a subformula of $\varphi$.

Assume that we have reached a position $(\mathfrak {A},t,\#,\lnot \psi )$ in a play of the game. The play of the game continues from the position $(\mathfrak {A},t,\overline{\#},\psi )$, where $\overline{\#}\in \{+,-\}\setminus \{\#\}$.

Assume a position $(\mathfrak {A},t,+,\psi \vee \psi ')$ has been reached. Then the player $\fancyscript{A}$ chooses exactly one of the sets $\{\psi ,\psi '\}$, $\{\psi \}$, $\{\psi '\}$. If $\fancyscript{A}$ chooses $\{\psi ,\psi '\}$, then $\fancyscript{I}$ chooses a formula $\chi \in \{\psi ,\psi '\}$, and the play continues from the position $(\mathfrak {A},t,+,\chi )$. If $\fancyscript{A}$ chooses $\{\psi \}$, then the play of the game continues from the position $(\mathfrak {A},t,+,\psi )$. If $\fancyscript{A}$ chooses $\{\psi '\}$, then the play continues from the position $(\mathfrak {A},t,+,\psi ')$.^6^ The background *intuition* concerning the disjunction rule is that $\fancyscript{A}$ makes one of the following three claims.

1. Both $\psi$ and $\psi '$ hold.
2. At least $\psi$ holds.
3. At least $\psi '$ holds.

If a position $(\mathfrak {A},t,-,\psi \vee \psi ')$ has been reached, the player $\fancyscript{I}$ chooses one of the positions $(\mathfrak {A},t,-,\psi )$ and $(\mathfrak {A},t,-,\psi ')$. The play of the game then continues from the position chosen by $\fancyscript{I}$.

Assume we have reached a position $(\mathfrak {A},t,+,Qx\, \psi )$ in the game, where *Q* is an ordinary generalized quantifier of type $(1)$. The play of the game continues as follows.

1. In the case $Q^{\mathfrak {A}}$ is empty, the play ends in the position $(\mathfrak {A},t,+, Qx\, \psi )$, and we say that the player $\fancyscript{A}$*does not survive* the play of the game. Otherwise, the player $\fancyscript{A}$ chooses a set $S\in Q^{\mathfrak {A}}$. The background *intuition* is that $\fancyscript{A}$ claims that *S* is the set of *exactly all* values $a\in A$ such that $t[\, x/a\, ]$ verifies the formula $\psi$.
2. Then the player $\fancyscript{I}$ chooses either the set *S* chosen by $\fancyscript{A}$ or its complement $A\setminus S$.
  - If $\fancyscript{I}$ chooses *S*, then $\fancyscript{I}$ also chooses an element $b\in S$, and the play of the game continues from the position $(\mathfrak {A},t[x/b],+,\psi )$. In this case the intuition is that the player $\fancyscript{I}$ is opposing the claim that *b* verifies $\psi$. If $S=\emptyset$ and $\fancyscript{I}$ chooses *S*, the play of the game ends in the position $(\mathfrak {A},t,+, Qx\, \psi )$, and the player $\fancyscript{A}$*survives* the play of the game.
  - If $\fancyscript{I}$ chooses $A\setminus S$, then $\fancyscript{I}$ also chooses an element $b\in A\setminus S$. The play of the game continues from the position $(\mathfrak {A},t[x/b],-,\psi )$. The intuition is that the player $\fancyscript{I}$ is opposing the claim that *bfalsifies*$\psi$. If $\fancyscript{I}$ chooses the set $A\setminus S$ and $A\setminus S =\emptyset$, the play of the game ends in the position $(\mathfrak {A},t,+, Qx\, \psi )$, and the player $\fancyscript{A}$*survives* the play of the game.

Assume we have reached a position $(\mathfrak {A},t,-,Qx\, \psi )$ in a play of the game, where *Q* is an ordinary generalized quantifier of type $(1)$. The play continues as follows.

1. If $\overline{Q}^{\mathfrak {A}}$ is empty, the play of the game ends in the position $(\mathfrak {A},t,-, Qx\, \psi )$, and the player $\fancyscript{A}$ does *not survive* the play of the game. Otherwise, the player $\fancyscript{A}$ chooses a set $S\in \overline{Q}^{\mathfrak {A}}$. The *intuition* is that the player $\fancyscript{A}$ claims that *S* is the set of *exactly all* values for *x* that verify $\psi$, and furthermore, $S\not \in Q^{\mathfrak {A}}$.
2. The player $\fancyscript{I}$ then chooses either the set *S* chosen by $\fancyscript{A}$ or its complement $A\setminus S$.
  - If $\fancyscript{I}$ chooses *S*, then $\fancyscript{I}$ also chooses an element $b\in S$, and the play of the game continues from the position $(\mathfrak {A},t[x/b],+,\psi )$. In this case the intuition is that the player $\fancyscript{I}$ is opposing the claim that *b* verifies $\psi$. If $\fancyscript{I}$ chooses *S* and $S=\emptyset$, the play of the game ends ends in the position $(\mathfrak {A},t,-, Qx\, \psi )$, and the player $\fancyscript{A}$ survives the play of the game.
  - If $\fancyscript{I}$ chooses $A\setminus S$, then $\fancyscript{I}$ also chooses an element $b\in A\setminus S$. The game continues from the position $(\mathfrak {A},t[x/b],-,\psi )$. The intuition is that the player $\fancyscript{I}$ is opposing the claim that *b* falsifies $\psi$. If $\fancyscript{I}$ chooses $A\setminus S$ and $A\setminus S=\emptyset$, the play ends in the position $(\mathfrak {A},t,-, Qx\, \psi )$, and the player $\fancyscript{A}$ survives the play of the game.

Assume we have reached a position $(\mathfrak {A},t,+, Mx\, \psi )$ in the game, where *M* is a minor quantifier. The play of the game continues as follows.

1. In the case $M^{\mathfrak {A}}$ is empty, the play ends in the position $(\mathfrak {A},t,+, Mx\, \psi )$, and we say that the player $\fancyscript{A}$*does not survive* the play of the game. Otherwise, the player $\fancyscript{A}$ chooses a pair $(S,T)\in M^{\mathfrak {A}}$. The *intuition* is that *S* and *T* are sets of values for *x*, witnessing and falsifying $\psi$, respectively. In other words, the player $\fancyscript{A}$ claims that assignments in $t[x/S]$ satisfy $\psi$, while assignments in $t[x/T]$ falsify $\psi$. A further piece of the background *intuition* of course is that providing such a pair $(S,T)$ is sufficient for the verification of $Mx\, \psi$.
2. Then the player $\fancyscript{I}$ chooses either the set *S* or the set *T*.
  - If $\fancyscript{I}$ chooses *S*, then $\fancyscript{I}$ also chooses an element $b\in S$, and the play of the game continues from the position $(\mathfrak {A},t[x/b],+,\psi )$. In this case the intuition is that the player $\fancyscript{I}$ is opposing the claim that *b* verifies $\psi$. If $S=\emptyset$ and $\fancyscript{I}$ chooses *S*, the game ends in the position $(\mathfrak {A},t,+,Mx\, \psi )$, and the player $\fancyscript{A}$ survives the play of the game.
  - If $\fancyscript{I}$ chooses *T*, then $\fancyscript{I}$ also chooses an element $b\in T$. The play of the game continues from the position $(\mathfrak {A},t[x/b],-,\psi )$. The intuition is that the player $\fancyscript{I}$ is opposing the claim that *b* falsifies $\psi$. If $T =\emptyset$ and $\fancyscript{I}$ chooses *T*, the game ends in the position $(\mathfrak {A},t,+,Mx\, \psi )$, and the player $\fancyscript{A}$ survives the play of the game.

Assume we have reached a position $(\mathfrak {A},t,-,Mx\, \psi )$ in a play of the game, where *M* is a minor quantifier. The play continues as follows.

1. In the case $\overline{M}^{\mathfrak {A}}$ is empty, the play of the game ends in the position $(\mathfrak {A},t,-, Mx\, \psi )$, and the player $\fancyscript{A}$ does not survive the play of the game. Otherwise, the player $\fancyscript{A}$ chooses a pair $(S,T)\in \overline{M}^{\mathfrak {A}}$. The intuition is that that *S* and *T* are sets of values witnessing and falsifying $\psi$, respectively, and supplying such a pair $(S,T)$ is enough to falsify $Mx\, \psi$.
2. The player $\fancyscript{I}$ then chooses either the set *S* or the set *T*.
  - If $\fancyscript{I}$ chooses *S*, then $\fancyscript{I}$ also chooses an element $b\in S$, and the play of the game continues from the position $(\mathfrak {A},t[x/b],+,\psi )$. The intuition is that the player $\fancyscript{I}$ is opposing the claim that *b* verifies $\psi$. If $\fancyscript{I}$ chooses *S* and $S = \emptyset$, the play of the game ends in the position $(\mathfrak {A},t,-,Mx\, \psi )$, and the player $\fancyscript{A}$ survives the play of the game.
  - If $\fancyscript{I}$ chooses *T*, then $\fancyscript{I}$ also chooses an element $b\in T$. The game continues from the position $(\mathfrak {A},t[x/s],-,\psi )$. The intuition is that the player $\fancyscript{I}$ is opposing the claim that *b* falsifies $\psi$. If $\fancyscript{I}$ chooses *T* and $T = \emptyset$, the play of the game ends in the position $(\mathfrak {A},t,-,Mx\, \psi )$, and the player $\fancyscript{A}$ survives the play of the game.

If $\psi$ is an atomic first-order formula and a position $(\mathfrak {A},t,+,\psi )$ is reached in a play of the game, then $\fancyscript{A}$ survives the play of the game if $\mathfrak {A},t\models _{\mathrm{FO}}\psi$. If $\mathfrak {A},t\not \models _{\mathrm{FO}}\psi$, then $\fancyscript{A}$ does not survive the play. If a position $(\mathfrak {A},t,-,\chi )$ is reached, where $\chi$ is an atomic first-order formula, then $\fancyscript{A}$ survives the play of the game if $\mathfrak {A},t\not \models _{\mathrm{FO}}\chi$. If $\mathfrak {A},t\models _{\mathrm{FO}}\chi$, then $\fancyscript{A}$ does not survive the play. If a position $(\mathfrak {A},t,+,\psi )$ or $(\mathfrak {A},t,-,\psi )$ is reached, where $\psi$ is a generalized atom, then $\fancyscript{A}$ survives the play. When a position with an atomic formula is reached, the play of the game ends in that position.

Let *U* and *V* be teams with the same domain. Assume the domain contains the free variables of $\varphi$. A play of the game $G(\mathfrak {A},U,V,\varphi )$ is played by $\fancyscript{A}$ and $\fancyscript{I}$ such that $\fancyscript{I}$ picks a beginning position $(\mathfrak {A},s,+,\varphi )$ or $(\mathfrak {A},t,-,\varphi )$, where $s\in U$ and $t\in V$. The play then proceeds according to the rules discussed above. If $U = V = \emptyset$ and therefore $\fancyscript{I}$ cannot choose a beginning position, then $\fancyscript{A}$ survives the unique play of the game. In this case no *end position* in the play of the game is generated.

Let *F* be a strategy of $\fancyscript{A}$ for the game $G(\mathfrak {A},U,V,\varphi )$; a strategy of $\fancyscript{A}$ is simply a function that provides a unique choice for $\fancyscript{A}$ in every possible position of the game that requires a choice by $\fancyscript{A}$. The domain of *F* is the set of positions in the game $G(\mathfrak {A},U,V,\varphi )$ that can be reached in some play of the game and require a choice by $\fancyscript{A}$. In a position $(\mathfrak {A},t,+,Kx\, \psi )$, if $K^{\mathfrak {A}}$ is empty, then the function *F* is undefined on the input $(\mathfrak {A},t,+,Kx\, \psi )$. Hence *F* does not provide any move for $\fancyscript{A}$ in such a position. Similarly, in a position $(\mathfrak {A},t,-,Kx\, \psi )$, if $\overline{K}^{\mathfrak {A}}$ is empty, then the function *F* is undefined on the input $(\mathfrak {A},t,-,Kx\, \psi )$. Here *K* can be a minor quantifier or an ordinary generalized quantifier.

Consider an atomic formula $\chi$. Let *S* be the set of assignments *t* such that some play, where $\fancyscript{A}$ plays according to the strategy *F*, ends in the position $(\mathfrak {A},t,+,\chi )$. The set *S* is the *team of positive final assignments* of the formula $\chi$ in the game $G(\mathfrak {A},U,V,\varphi )$, when $\fancyscript{A}$ plays according to *F*. Similarly, let *T* be the set of assignments *t* such that some play, where $\fancyscript{A}$ plays according to *F*, ends in the position $(\mathfrak {A},t,-,\chi )$. The set *T* is the *team of negative final assignments* of the formula $\chi$ in the game $G(\mathfrak {A},U,V,\varphi )$, when $\fancyscript{A}$ plays according to *F*.

A *survival strategy* of $\fancyscript{A}$ in a game $G(\mathfrak {A},U,V,\varphi )$ is a strategy that guarantees, in every play of the game where $\fancyscript{A}$ follows *F*, a survival for $\fancyscript{A}$. Let *F* be a survival strategy for $\fancyscript{A}$ in $G(\mathfrak {A},U,V,\varphi )$. Let $S(\chi )$ and $T(\chi )$ denote, respectively, the teams of positive and negative final assignments of the generalized atom $\chi$ in the game $G(\mathfrak {A},U,V,\varphi )$, when $\fancyscript{A}$ plays according to *F*. The survival strategy *F* is a *uniform survival strategy* for $\fancyscript{A}$, if for every generalized atom $\chi$ in $\varphi$, we have $\mathfrak {A},\bigl (S(\chi ),T(\chi )\bigr )\models \chi$.

Recall that all occurrences of a subformula in a formula $\varphi$ are considered to be distinct subformulae of $\varphi$. Therefore, for example, if $\varphi$ is a generalized atom and a game $G(\mathfrak {A},U,V,\varphi \vee \varphi )$ is played according to some strategy, the teams of final assignments for the different instances of $\varphi$ may turn out different.

When $\mathfrak {A}$ is known from the context, we sometimes write $G(U,V,\psi )$ instead of $G(\mathfrak {A},U,V,\psi )$. Also, we may write $(s,\#,\psi )$ instead of $(\mathfrak {A},s,\#,\psi )$.

### **Theorem 1**

$\mathfrak {A}, (U,V)\models \varphi$ iff there exists a uniform survival strategy for $\fancyscript{A}$ in the game $G(\mathfrak {A},U,V,\varphi )$.

### *Proof*

The claim is proved by induction on the structure of $\varphi$. The case for atomic formulae is trivial.

Assume that $(U,V)\models \lnot \psi$. Therefore $(V,U)\models \psi$. By the induction hypothesis, $\fancyscript{A}$ has a uniform survival strategy *F* in $G(V,U,\psi )$. The strategy *F* provides a uniform survival strategy in $G(U,V,\lnot \psi )$.

Assume that $\fancyscript{A}$ has a uniform survival strategy in $G(U,V,\lnot \psi )$. Therefore $\fancyscript{A}$ has a uniform survival strategy in $G(V,U,\psi )$. By the induction hypothesis, $(V,U)\models \psi$. Therefore $(U,V)\models \lnot \psi$.

Assume that $(U,V)\models \psi \vee \psi '$. Thus we have $(U_1,V)\models \psi$ and $(U_2,V)\models \psi '$ for some $U_1,U_2\subseteq U$ such that $U_1\cup U_2 = U$. By the induction hypothesis, the player $\fancyscript{A}$ has a uniform survival strategy $F_1$ in the game $G(U_1,V,\psi )$ and $F_2$ in the game $G(U_2,V,\psi ')$. Define a strategy *F* for $G(U,V,\psi \vee \psi ')$ such that

$$\begin{aligned} F\bigl (\, (s,+,\psi \vee \psi ')\, \bigr )= {\left\{ \begin{array}{ll} \{\psi ,\psi '\} &{} \hbox { if }\ s\in U_1\cap U_2\\ \{\psi \} &{} \hbox { if }\ s\in U_1\setminus U_2\\ \{\psi '\} &{} \hbox { if }\ s\in U_2\setminus U_1\\ \end{array}\right. } \end{aligned}$$

for each $s\in U$. On other positions, *F* agrees with $F_1$ or $F_2$, depending on whether the input position contains a subformula of $\psi$ or $\psi '$. Let $\chi$ be an atomic subformula of $\psi \vee \psi '$. If $\chi$ is a subformula of $\psi$, then the strategy *F* gives the same final team of assignments for $\chi$ as $F_1$. If, on the other hand, $\chi$ is a subformula of $\psi '$, then *FF* gives the same final team of assignments for $\chi$ as $F_2$. Therefore *F* is a uniform survival strategy for $\fancyscript{A}$ in $G(U,V,\psi \vee \psi ')$.

Assume there exists a uniform survival strategy *F* for $G(U,V,\psi \vee \psi ')$. Define $U_1\subseteq U$ to be the set of assignments $s\in U$ such that $F\bigl (\, (s,+,\psi \vee \psi ')\, \bigr )\, =\, \{\psi ,\psi '\}$ or $F\bigl (\, (s,+,\psi \vee \psi ')\, \bigr )\, =\, \{\psi \}$. Similarly, define $U_2\subseteq U$ to be the set of assignments $s\in U$ such that $F\bigl (\, (s,+,\psi \vee \psi ')\, \bigr )\, = \, \{\psi ,\psi '\}$ or $F\bigl (\, (s,+,\psi \vee \psi ')\, \bigr )\, =\, \{\psi '\}$. Now, *F* provides uniform survival strategies for $G(U_1,V,\psi )$ and for $G(U_2,V,\psi ')$. By the induction hypothesis, $(U_1,V)\models \psi$ and $(U_2,V)\models \psi '$. Since $U_1\cup \, U_2 = U$, we have $(U,V)\models \psi \vee \psi '$.

We shall not discuss the argument for ordinary generalized quantifiers, since the related details are essentially provided by the argument for minor quantifiers.

Assume that $(U,V)\models Mx\, \psi$. Thus there exists functions $f:U\rightarrow M^{\mathfrak {A}}$ and $g:V\rightarrow \overline{M}^{\mathfrak {A}}$ such that

$$\begin{aligned} \bigl (U[x/f]\cup V[x/g] ,U[x/f\, '\, ]\cup V[\, x/g'\, ]\bigr )\models \psi . \end{aligned}$$

By the induction hypothesis, there exists a uniform survival strategy *F* in

$$\begin{aligned} G\bigl (U[x/f]\cup V[x/g] ,U[x/f\, '\, ]\cup V[\, x/g'\, ],\psi \bigr ). \end{aligned}$$

Extend the strategy *F* to a strategy $F^+$ such that $F^+\bigl (\, (s,+,Mx\, \psi )\, \bigr )\, =\, f(s)$ for each $s\in U$ and $F^+\bigl (\, (t,-,Mx\, \psi )\, \bigr )\, =\, g(t)$ for each $t\in V$. The strategy $F^+$ gives the same final teams of assignments as *F*, and hence $F^+$ is a uniform survival strategy for $\fancyscript{A}$ in $G(U,V,Mx\, \psi )$.

Assume *F* is a uniform survival strategy in $G(U,V,Mx\, \psi )$. Define the function $f:U\rightarrow M^{\mathfrak {A}}$ such that $f(s)= F\bigl (\, (s,+,Mx\, \psi )\, \bigr )$ for all $s\in U$. Define also the function $g:V\rightarrow \overline{M}^{\mathfrak {A}}$ such that $g(s) = F\bigl (\, (s,-,Mx\, \psi )\, \bigr )$ for all $s\in V$. Now, *F* provides a uniform survival strategy for

$$\begin{aligned} G\bigl (U[x/f]\cup V[x/g],U[x/f\, '\, ]\cup V[\, x/g\, '\, ],\psi \bigr ). \end{aligned}$$

By the induction hypothesis,

$$\begin{aligned} \bigl (U[x/f]\cup V[x/g],U[x/f\, '\, ]\cup V[\, x/g\, '\, ]\bigr )\models \psi . \end{aligned}$$

Therefore $$(U,V)\models Mx\, \psi $$.$$\square $$

## Interpreting Dependence Logic with Double Team Semantics

In this section we discuss a simple natural way of interpreting variants of dependence logic with double team semantics. We also address some issues concerning the interpretation of dependence logic and its variants.

Let *k* be a positive integer and *T* a non-empty set. Let $R\subseteq T^k$ be a relation. We say that *Ris a partial function*, if the following conditions hold.

1. If $$k = 1$$, then $$| R | \le 1$$.
2. If $k>1$, and if we have $(b_1,\ldots ,b_{k-1},c)\in R$ and $(b_1,\ldots ,b_{k-1},d)\in R$, then $c = d$.

For each positive integer *k*, let $\fancyscript{D}_k$ denote the generalized quantifier of type $(k,k)$ such that $\fancyscript{D}_k$ contains the triples $(A,R,S)$ such that the following conditions hold.

1. *A* is a nonempty set.
2. $$R\subseteq A^k$$ and $$S\subseteq A^k$$.
3. *R* is a partial function and $S = \emptyset$.

Let $\varDelta$ be the class $\{\ \fancyscript{D}_k\ |\ k\in \mathbb {Z}_+\ \}$.

We next define a translation of formulae of dependence logic $\hbox {D}$ into a logic with the minor quantifier $\exists ^s$ and generalized atoms $D_k(x_1,\ldots ,x_k;x_1,\ldots ,x_k)$ for each $k\in \mathbb {Z}_+$; the semantics of the atom $D_k(x_1,\ldots ,x_k;x_1,\ldots ,x_k)$ is given by the generalized quantifier $\fancyscript{D}_k\in \varDelta$. Define the following translation function *T*:

1. If $\varphi$ is a first-order atom, then $T(\varphi ) = \varphi$ and $T(\lnot \varphi ) = \lnot \varphi$.
2. $$T\bigl (=\!\!(x_1,\ldots ,x_k)\bigr )= D_k(x_1,\ldots ,x_k;x_1,\ldots ,x_k)$$.
3. $$T\bigl (\lnot =\!\!(x_1,\ldots ,x_k)\bigr )= \lnot D_k(x_1,\ldots ,x_k ;x_1,\ldots ,x_k)$$.
4. $$T(\varphi \vee \psi ) = \bigl (T(\varphi )\vee T(\psi )\bigr )$$.
5. $$T(\varphi \wedge \psi ) = \lnot \bigl (\lnot T(\varphi )\vee \lnot T(\psi )\bigr )$$.
6. $$T(\exists z\, \varphi ) = \exists ^s z\, \varphi $$.
7. $$T(\forall z\, \varphi ) = \lnot \, \exists ^s z\, \lnot \, T(\varphi )$$.

The following proposition is immediate.

### **Proposition 3**

Let $\varphi$ be a formula of dependence logic. Then $\mathfrak {A}\models _{U}\varphi$ iff $\mathfrak {A},(U,\emptyset )\models T(\varphi )$.

Obviously inclusion logic with strict semantics can be similarly translated into a logic with double team semantics. A different class of generalized quantifiers is needed in order to define the atoms that inclusion atoms translate to, and the alternative disjunction $\vee ^s$ defined in Sect. 4 is used in the target language. Also inclusion logic with lax semantics can be analogously translated. Standard disjunctions are used in the target language, and existential quantifiers translate to the lax quantifier $\exists ^l$.

### Interpreting Different Existential Quantifiers

It is interesting to note that neither the strict nor the lax existential quantifier is the same as the minor quantifier $M_{\exists }$ defined by the existential quantifier. It is natural to consider the three different existential quantifiers as *epistemic variants* of each other. Let us briefly discuss what this perspective implies.

Consider the game-theoretic semantics for minor quantifiers. Let $\varphi (x)$ be a first-order formula. To show that the formula $\exists ^s x\,\varphi (x)$ is true, the agent $\fancyscript{A}$ simply has to find a single witness *b* such that the formula $\varphi (b)$ holds. It is enough that the agent *knows* one suitable witness *b* for $\varphi (x)$.

Let $\exists ^t$ denote the minor quantifier $M_{\exists }$, and call it the *total existential quantifier*. Establishing that $\exists ^t x\, \varphi (x)$ holds is rather different from showing that $\exists ^s x\, \varphi (x)$ is true. This time it is not enough for the agent to know a single witness for $\varphi (x)$. Instead, the agent has to be able to say, for each element *b* in the domain of the model under investigation, whether $\varphi (b)$ holds or not. Therefore the agent has to have an *epistemically complete understanding* of which elements of the domain satisfy $\varphi (x)$ and which ones do not.

Indeed, the strict existential quantifier seems to correspond to the intuitive understanding of *ordinary* existence claims better than the total existential quantifier. But of course $\exists ^t$ may be more appropriate than $\exists ^s$ in some non-standard context.

Establishing that $\exists ^l x\, \varphi (x)$ is similar to showing that $\exists ^s x\, \varphi (x)$, but here the agent can provide more than one witness to be taken into account in the rest of the semantic game.

In light of Propositions 2 and 1, the three existential quantifiers are interchangeable in the context of ordinary first-order logic. But it is possible to conceive natural non-classical logics—possibly dealing with epistemic considerations, and not necessarily involving generalized atoms—where different epistemic modes of existential quantification make a crucial difference. And, it is not difficult to define ad hoc atoms $A(x;x)$ such that, say, the formulae $\exists ^s x\, A(x;x)$, $\exists ^l x\, A(x;x)$, $\exists ^t x\, A(x;x)$ are pairwise non-equivalent.

Indeed, define the atom $A(x;x)$ such that $\mathfrak {A},(U,V)\models A(x;x)$ if and only if we have $|{Rel}(U,x)| = 2$ and ${Rel}(V,x) = \emptyset$. Let $\mathfrak {B}$ be a model whose domain contains exactly two elements. Now $\mathfrak {B}\models \exists ^l x A(x;x)$, $\mathfrak {B}\models \exists ^t x A(x;x)$, but $\mathfrak {B}\not \models \exists ^s x A(x;x)$. Now let $\mathfrak {C}$ be a model whose domain contains exactly three elements. We have $\mathfrak {C}\models \exists ^l x A(x;x)$, while $\mathfrak {C}\not \models \exists ^t x A(x;x)$. Therefore the formulae $\exists ^s x\, A(x;x)$, $\exists ^l x\, A(x;x)$, $\exists ^t x\, A(x;x)$ are pairwise non-equivalent.

Let *T* denote the trivial generalized quantifier of type $(1)$ defined such that $\mathfrak {A}\models _{\mathrm{FO}} Tx\, \varphi$ always holds. In the double team framework, the statement $\mathfrak {A}, (\{\emptyset \}, \emptyset ) \models Tx\, P(x)$ means that the player $\fancyscript{A}$ can *classify* all elements $b\in {Dom}(\mathfrak {A})$ according to whether $P(b)$ holds or not, i.e., $\fancyscript{A}$ can point out exactly the set of values *b* such that $P(b)$. The statement $\mathfrak {A}, (\{\emptyset \}, \emptyset ) \models \exists ^t\, x\, P(x)$ means that the player $\fancyscript{A}$ can classify all elements *b* of the domain of $\mathfrak {A}$ according to whether $P(b)$ holds or not, and the set of values such that $P(b)$ holds, is nonempty. These are constructive statements that clearly *differ from the ordinary reading* of the generalized quantifiers *T* and $\exists$. The notion of a minor quantifier provides a novel way of generalizing the notion of a generalized quantifier by providing a fine-grained picture of constructive issues related to verification of quantified formulae.

A possible future research direction could include considering semantic games where choosing (sets of) witnesses would be associated with a *cost*, and of course the player(s) involved would have limited amounts of resources with which to meet the costs. For example, in a very simple case, each element of the domain of a model could be associated with a unit cost. Such games could help in the analysis of *proving or verifying theorems with limited resources*. The article Kuusisto (2010) describes a related tentative *resource conscious* approach to the semantics of first-order logic. In that article, verifying the formula $\exists x P(x)$ requires a single witness that satisfies the formula $P(x)$. Thus the existential quantifier there corresponds to $\exists ^s$. The article does not consider alternative existential quantifiers, but it would indeed be interesting to define a resource conscious semantics also for the total quantifier $\exists ^t$. We leave these investigations for the future.

### Observations Concerning Atoms

Above we translated dependence atoms $=\!\!(x_1,\ldots ,x_k)$ into atomic expressions

$$\begin{aligned} D_k(x_1,\ldots ,x_k; x_1,\ldots ,x_k). \end{aligned}$$

This creates an unnecessary syntactic complication: it seems rather pointless to write $x_1,\ldots ,x_k$ twice. We can of course avoid such complications in similar translations simply by allowing for syntactic atomic expressions $A(x_1,\ldots ,x_k)$, whose semantics is defined by a generalized quantifier of type $(k,k)$, and more generally, atoms $B(\overline{x}_1,\ldots ,\overline{x}_k)$ defined by quantifiers of type $(i_1,\ldots ,i_k,i_1,\ldots ,i_k)$.

Let $(Q,P)$ be a pair of generalized quantifiers of type $(i_1,\ldots ,i_k)$. Consider atomic expressions of type $B(\overline{x}_1,\ldots ,\overline{x}_k),$ where each tuple $\overline{x}_j$ is of length $i_j$. Extend the double team semantics such that $\mathfrak {A},(U,V)\models B(\overline{x}_1,\ldots ,\overline{x}_k)$ iff

$$\begin{aligned}&\bigl (\hbox {Rel}(U,\mathfrak {A},\overline{x}_1),\ldots , \hbox {Rel}(U,\mathfrak {A},\overline{x}_k)\bigr )\in Q^{\mathfrak {A}}\\ \hbox {and}&\\&\bigl (\hbox {Rel}(V,\mathfrak {A},\overline{x}_{1}),\ldots , \hbox {Rel}(V,\mathfrak {A},\overline{x}_{k})\bigr )\in P^{\, \mathfrak {A}}. \end{aligned}$$

If $P = \overline{Q}$, we call the atom defined by $(Q,P)$ a *symmetric atom*.

It is interesting to note that above it would *not* have been possible to translate atoms $=\!\!(x_1,\ldots ,x_k)$ to symmetric atoms $B(x_1,\ldots ,x_k)$. The truth definitions of the dependence atom $=\!\!(x_1,\ldots ,x_k)$ and its negated counterpart $\lnot =\!\!(x_1,\ldots ,x_k)$ are not related in a way that would lead to the required symmetry.

Currently, there does not seem to be an account in the dependence logic literature that thoroughly analyzes issues related to the definition $\mathfrak {A}\models _{U}\lnot =\!\!(x_1,\ldots ,x_k)$ iff $U = \emptyset$. It is well known that dependence logic is downwards closed, i.e., if $\mathfrak {A}\models _U\varphi$ and $V\subseteq U$, then $\mathfrak {A}\models _V\varphi$. The definition $\mathfrak {A}\models _{U}\lnot =\!\!(x_1,\ldots ,x_k)\ \Leftrightarrow \ \mathfrak {A}\not \models _{U}\, =\!\!(x_1,\ldots ,x_k)$ would lead to a logic that is not downwards closed. Downwards closure is a natural intuitive property of dependence logic. The property reflects the background *intuition* that a team satisfies a formula if all assignments in the team satisfy it.^7^ With the semantics $\mathfrak {A}\models _U\lnot =\!\!(x_1,\ldots ,x_k)\ \Leftrightarrow \ U =\emptyset$ for negated dependence atoms, dependence logic is downwards closed, but still this choice of definition may perhaps seem intuitively somewhat strange. At least the definition calls for further reflection.

In inclusion logic (see Galliani 2012), negated non-first-order atoms are not allowed, and thereby no analogous problem of interpretation arises. However, the possibility of negating atomic formulae—a syntactically rather natural feature—is compromised.

We shall not attempt to analyze the issue concerning negated atoms further, but we wish to point out that the double team framework can perhaps help in advancing the *interpretation* of formalisms in the family of dependence logic for the following three reasons.

Firstly, the double team semantics provides a *general* framework for interpreting different variants of dependence logic. A general framework offers a setting for interpreting and *comparing* different systems embeddable in the framework. For example, we have above given natural possible interpretations for the quantifiers $\exists ^s$, $\exists ^l$, $\exists ^t$ and discussed their differences in the interpreting framework.

Secondly, the double team semantics has obvious *symmetric* duality properties concerning the interpretation of negation. Whatever the explanatory power of symmetries may be, at least symmetric duality properties have an obvious mathematical appeal.

Finally, the double team semantics has a very natural game-theoretic counterpart. A game-theoretic semantics can—at least in some reasonable sense—be seen as *fundamental* in relation to most other approaches, because it provides an *action-based* account of the meaning of formulae. Tarski’s semantics for first-order logic essentially only gives a *translation* of symbols into their natural language counterparts.^8^ This resembles translating a language into another. An interpreter has to be familiar with the target language in order to understand the truth definition. The situation seems different in the context of action-based truth definitions.^9^ Action-based language acquisition is discussed for example in Steels and Kaplan (2001). Wittgenstein’s *language games*, described in Wittgenstein (1953), are a classical example of related considerations.

## Complexity of $\hbox {DC}^2$

### The Logic $\hbox {DC}^2$

In this section we define the logic $\hbox {DC}^2$. This logic extends both ordinary two variable dependence logic $\hbox {D}^2$ and two-variable logic with counting $\hbox {FOC}^2$ in a canonical way, as we shall see.

Let *k* be a positive integer. Define the classes

$$\begin{aligned} \fancyscript{E}\, :=\, \{\, (A,B,\emptyset )\ |\ A\,\hbox {is a non-empty set and}\,B\subseteq A\,\hbox {satisfies}\,| B |\ge k\ \} \end{aligned}$$

and

$$\begin{aligned} \fancyscript{F}\, :=\, \{\, (A,\emptyset ,B)\ |\ A\hbox { is a non-empty set,} \,B\subseteq A\,\hbox {and,}\,| A\setminus B | < k\ \}. \end{aligned}$$

The pair $(\fancyscript{E},\fancyscript{F})$ defines the *minor counting quantifier*$\exists ^{\ge k}$. Notice that $\exists ^{{\ge k}}$ is a minor of the generalized quantifier $\{\ (A,B)\ |\ A\not =\emptyset ,\ |B|\ge k\ \}$.

Let $\tau$ be a relational vocabulary consisting of the union of a countably infinite set of unary relation symbols and a countably infinite set of binary relation symbols. Fix two distinct first-order variable symbols *x* and *y*. Define $\fancyscript{A}(\tau )$ to be the smallest set *T* such that the following conditions hold.

1. If $P\in \tau$ is a unary relation symbol and $z\in \{x,y\}$, then $P(z)\in T$.
2. If $R\in \tau$ is a binary relation symbols and $z,z'\in \{x,y\}$, then $R(z,z')\in T$.
3. If $$z,z'\in \{x,y\}$$, then $$z = z'\in T$$.

Define *two-variable first-order logic with counting*$(\hbox {FOC}^2)$ to be the smallest set *T* such that the following conditions are satisfied.

1. $$\fancyscript{A}(\tau )\subseteq T$$.
2. If $$\varphi \in T$$, then $$\lnot \varphi \in T$$.
3. If $$\varphi ,\psi \in T$$, then $$(\varphi \vee \psi )\in T$$.
4. If $\varphi \in T$, $z\in \{x,y\}$, and *k* is a positive integer, then $\exists ^{\ge k} z\, \varphi \in T$.

Here $\exists ^{\ge k}$ denotes the minor quantifier $\bigl (\fancyscript{E},\fancyscript{F}\bigr )$. The syntax of $\hbox {FOC}^2$ contains only first-order atoms, and in light of Propositions 2 and 1, it makes no difference whether we use ordinary Tarskian semantics or double team semantics in the interpretation of $\hbox {FOC}^2$-formulae; if $\varphi$ is a formula of $\hbox {FOC}^2$ and $\varphi '$ denotes the formula obtained from $\varphi$ by replacing each symbol $\exists ^{\ge k}$ by a symbol that denotes the corresponding ordinary generalized quantifier, then $\mathfrak {A},s\models _{\mathrm{FO}}\varphi '$ iff $\mathfrak {A},\bigl (\{s\},\emptyset \bigr )\models \varphi$.

Define $\fancyscript{A}^+(\tau )$ to be the smallest set *T* such that the following conditions hold.

1. If $$\varphi \in \fancyscript{A}(\tau )$$, then $$\varphi \in T$$.
2. If $z,z'\in \{x,y\}$, then $=\!\!(z,z')\in T$ and $=\!\!(z)\in T$.

The set of formulae of $\hbox {DC}^2$ is the smallest set *T* such that the following conditions hold.

1. $$\fancyscript{A}^+(\tau )\subseteq T$$.
2. If $$\varphi \in T$$, then $$\lnot \varphi \in T$$.
3. If $$\varphi ,\psi \in T$$, then $$(\varphi \vee \psi )\in T$$.
4. If $\varphi \in T$ and $z\in \{x,y\}$, then $\exists ^{s} z\, \varphi \in T$.
5. If $\varphi \in T$, $z\in \{x,y\}$ and *k* is a positive integer, then $\exists ^{\ge k} z\, \varphi \in T$.

Let $z,z'\in \{x,y\}$ be variables. The semantics of the atom $=\!\!(z)$ in is defined in $\hbox {DC}^2$ such that $\mathfrak {A},(U,V)\models \, =\!\!(z)$ iff $\mathfrak {A},(U,V)\models D_1(z;z)$. Similarly, $\mathfrak {A},(U,V)\models \, =\!\!(z,z')$ iff $\mathfrak {A},(U,V)\models D_2\bigl ((z,z');(z,z')\bigr )$.

The following lemma is trivial.

#### **Lemma 1**

Let $(U,V)$ and $(S,T)$ be double teams such that $S\subseteq U$ and $T\subseteq V$.

Let $\varphi \in \fancyscript{A}^+(\tau )$ be any atomic formula of  $\hbox {DC}^2$.

If $$(U,V)\models \, \varphi $$, then $$(S,T)\models \, \varphi $$.

Obviously $\hbox {FOC}^2$ is contained in $\hbox {DC}^2$, but also $\hbox {D}^2$ is essentially contained in $\hbox {DC}^2$ via the translation *T* defined in Sect. 8 (see Proposition 3).

We have somewhat blindly copied the atoms of $\hbox {D}$ into $\hbox {DC}^2$; it is an interesting question what these atoms *exactly mean* in $\hbox {DC}^2$, and what other kinds of atoms and quantifiers should be considered. We leave such questions for the future. Our objective in the rest of the current article is simply to show how the double team semantics nicely facilitates the $\hbox {NEXPTIME}$-completeness proof of the logic $\hbox {DC}^2$ and other sufficiently similar logics.

### $\hbox {DC}^2$ is NEXPTIME-Complete

An input to the satisfiability or finite satisfiability problem of $\hbox {DC}^2$ is any sentence $\varphi$ of $\hbox {DC}^2$. Note that the set of non-logical symbols of $\varphi$ is limited to unary and binary relation symbols only. The satisfiability problem asks whether there exists a model $\mathfrak {A}$ such that $\mathfrak {A},\bigl (\{\emptyset \},\emptyset \, \bigr )\models \varphi$, while the finite satisfiability asks whether there exists a *finite* model $\mathfrak {B}$ such that $\mathfrak {B},\bigl (\{\emptyset \},\emptyset \, \bigr )\models \varphi$.

An input to the satisfiability or finite satisfiability problem of $\hbox {FOC}^2$ is any sentence $\varphi$ of $\hbox {FOC}^2$; the set of non-logical symbols of $\varphi$ is here limited to unary and binary relation symbols only. The satisfiability problem asks whether there exists a model $\mathfrak {A}$ such that $\mathfrak {A}\models _{\mathrm{FO}}\varphi$, while the finite satisfiability problem asks whether there exists a finite model $\mathfrak {B}$ such that $\mathfrak {B}\models _{\mathrm{FO}}\varphi$.

Below we show that the satisfiability and finite satisfiability problems of $\hbox {DC}^2$ are $\hbox {NEXPTIME}$-complete. Our proof uses the fact that the satisfiability and finite satisfiability problems of $\hbox {FOC}^2$ are $\hbox {NEXPTIME}$-complete (see Pratt-Hartmann 2005). We translate $\hbox {DC}^2$ formulae into equisatisfiable formulae of $\hbox {FOC}^2$ with a polynomial cost in the formula length. A formula $\varphi$ translates to a formula

$$\begin{aligned} \varphi ^*\ :=\ \psi _{initial}\ \wedge \ \bigwedge \limits _{\chi \ \in \ \hbox {SUB}_{\varphi }}\psi _{\chi }, \end{aligned}$$

which we define in detail below.

Each conjunct $\psi _{\chi }$ contains two fresh relation symbols $S_{\chi }$ and $T_{\chi }$. Intuitively, the pair $(S_{\chi },T_{\chi })$ encodes the double team $(U_{\chi },V_{\chi })$ that satisfies $\chi$ when $\varphi$ is evaluated in a model where $\varphi$ holds. If $\chi$ is not an atom, the formula $\psi _{\chi }$ also contains auxiliary formulae that describe how double teams evolve when $\varphi$ is evaluated. For example, if $\chi = \exists ^{s} x\, \alpha$, then $\psi _{\chi }$ describes how the double team $(U_{\chi },V_{\chi })$ gives rise to a double team $(U_{\alpha },V_{\alpha })$ that satisfies $\alpha$.

In addition to relation symbols $S_{\chi }, T_{\chi }$ corresponding to double teams, further fresh variable symbols are used in $\psi _{\chi }$ when $\chi$ is a formula whose main connective is a quantifier. The fresh symbols $E_{\alpha }^{{ Uf}},\, E_{\alpha }^{{ Vg}'}$ correspond to the teams $U[z/f],\, V[z/g']$ needed in the truth definition of quantified formulae.^10^

The logic $\hbox {FOC}^2$ uses only two variables, and this creates some obstacles that need to be overcome when writing the formulae $\psi _{\chi }$. Due to the expressivity limitations of $\hbox {FOC}^2$, we need to control the evaluation of double teams $(U_{\chi },V_{\chi })$. For example, if $\chi = \exists ^{s} x\, \alpha$ and the domain of $U_{\chi }$ contains *x*, then we need to ensure that the new values of *x* in $U[ x/f ]$ are in a sense *independent* of the old values of *x* in $U_{\chi }$; the related definitions are formally discussed below. Lemma 2 ensures that we can indeed control the evaluation of the teams $(U_{\chi },V_{\chi })$ in the desired way, and therefore the two-variable logic $\hbox {FOC}^2$ is sufficiently expressive for our purposes.

While formulae $\psi _{\chi }$ describe double teams corresponding to subformulae of $\varphi$, the formula $\psi _{initial}$ simply sets the stage by asserting that the team satisfying $\varphi$ itself corresponds to the double team $\bigl (\{\emptyset \},\emptyset \, \bigr )$.

We are now ready for the formal details of the proof that the logic $\hbox {DC}^2$ is complete for $\hbox {NEXPTIME}$. We begin by some auxiliary definitions and the auxiliary Lemmata 2 and 3. We then formally define the conjuncts of $\varphi ^*$ and show that $\varphi$ and $\varphi ^*$ are equisatisfiable.

Let *U* be a team for a model $\mathfrak {A}$. Let *A* be the domain of $\mathfrak {A}$. Let $s,t\in U$ be assignments such that $s(z) = t(z)$ for all $z\in {Dom}(U)\setminus \{x\}$. Then *t* is called an *x-variant of s* (in *U*). Note that *s* is an *x*-variant of itself.

Let *M* be a minor quantifier, and let $N\in \{\, M^{\mathfrak {A}},\overline{M}^{\mathfrak {A}}\, \}$. Let $f:U\rightarrow N$ be a function. Assume that we have we have $f(s) = f(t)$ for all valuations $s,t\in U$ such that *t* is an *x*-variant of *s*. Then we say that *f* is *x-independent*. Let $g:U\rightarrow N$ be a function. Assume $g_0:U\rightarrow N$ is an *x*-independent function such that for each $s\in U$, there exists an *x*-variant $t\in U$ of *s* such that $g_0(s) = g(t)$. Then $g_0$ is an *x-independent minor of g*.

Let *U* be a team with domain $\{x,y\}$ and for a model $\mathfrak {A}$, where *x* and *y* are the variables used in $\hbox {DC}^2$ and $\hbox {FOC}^2$. We let ${Rel}(U)$ denote the relation ${Rel}\bigl (U,\mathfrak {A},(x,y)\bigr )$, as opposed to ${Rel}\bigl (U,\mathfrak {A},(y,x)\bigr )$. This means that we in a sense nominate *x* as the first variable and *y* as the second one. This convention will simplify the notation below. If *U* is a team with the domain $\{z\}$, where $z\in \{x,y\}$, then we let ${Rel}(U)$ denote ${Rel}\bigl (U,\mathfrak {A},z\bigr )$.

#### **Lemma 2**

Let $\psi$ be a formula of  $\hbox {DC}^2$. Let $M\in \{\exists ^{s},\exists ^{\ge k}\}$, where *k* is a positive integer. Let $z\in \{x,y\}$ be a variable. Let $f:U\rightarrow M^{\mathfrak {A}}$ and $g:V\rightarrow \overline{M}^{\mathfrak {A}}$ be functions, and let $f_0$ and $g_0$ be $z$-independent minors of *f* and *g*, respectively. If

$$\begin{aligned} \mathfrak {A},\bigl (U[z/f]\cup V[z/g],\, U[z/f\, '\, ]\cup V[\, z/g\, '\, ]\bigr )\models \psi , \end{aligned}$$

then

$$\begin{aligned} \mathfrak {A},\bigl (U[z/f_0]\cup V[z/g_0],\, U[z/f_0\, '\, ]\cup V[\, z/g_0\, '\, ]\bigr )\models \psi . \end{aligned}$$

#### *Proof*

Assume that

1 $$\begin{aligned} \bigl (U[z/f]\cup V[z/g],\, U[z/f\, '\, ]\cup V[\, z/g\, '\, ]\bigr )\models \psi . \end{aligned}$$

It is clear that $U[z/f_0]\subseteq U[z/f]$ and $V[z/g_0\, '\,]\subseteq V[\, z/g\, '\, ]$. It is also clear that $V[z/g_0\, ] = V[\, z/g\, ] = U[x/f_0\, '\, ] = U[x/f\, '\, ] = \emptyset$. Therefore

2 $$\begin{aligned} U[z/f_0]\cup V[z/g_0]\subseteq U[z/f]\cup V[z/g] \end{aligned}$$

and

3 $$\begin{aligned} U[z/f_0\, '\, ]\cup V[\, z/g_0\, '\, ] \subseteq U[z/f\, '\, ]\cup V[\, z/g\, '\, ]. \end{aligned}$$

We define a strategy for the player $\fancyscript{A}$ in the game

$$\begin{aligned} G^*\ :=\ G\bigl (\mathfrak {A},U[z/f_0]\cup V[z/g_0],U[z/f_0\, '\, ]\cup V[\, z/g_0\, '\, ],\psi \bigr ). \end{aligned}$$

Due to Eq. 1, player $\fancyscript{A}$ has a uniform survival strategy *F* in the game

$$\begin{aligned} G\ :=\ G\bigl (\mathfrak {A},U[z/f]\cup V[z/g],U[z/f\, '\, ]\cup V[\, z/g\, '\, ],\psi \bigr ). \end{aligned}$$

Due to Eqs. 2 and 3, the strategy *F* can be canonically restricted to a strategy *H* for the game $G^*$. We need to show that $H$ is a *uniform survival strategy* for $\fancyscript{A}$ in $G^*$.

Since *H* is a restriction of the uniform survival strategy *F*, the player $\fancyscript{A}$ survives each play of the game $G^*$ played according to *H*. To see that *H* is a uniform survival strategy, consider the sets $S^*(\chi )$ and $T^*(\chi )$ of positive and negative final assignments for an atomic subformula $\chi$ of $\psi$ in the game $G^*$ when $\fancyscript{A}$ follows *H*. Let $S(\chi )$ and $T(\chi )$ be the corresponding sets in the game *G* when $\fancyscript{A}$ follows *F*.

It is clear that $S^*(\chi )\subseteq S(\chi )$ and $T^*(\chi )\subseteq T(\chi )$. Due to Eq. 1, we have $\bigl (S(\chi ),T(\chi )\bigr )\models \chi$. By Lemma 1, we have $\bigl (S^*(\chi ),T^*(\chi )\bigr )\models \chi$, and therefore $H$ is a uniform survival strategy for $\fancyscript{A}$ in the game $G^*$. $\square$

Consider a logic where each atom $\chi$ satisfies the property that if $(U,V)\models \chi$,  $S\subseteq U$, and $T\subseteq V$, then we have $(S,T)\models \chi$. By analyzing the proof of Lemma 2, it is easy to see that we have actually proved that each such logic satisfies also the property that if $(U,V)\models \varphi$,  $S\subseteq U$, and $T\subseteq V$, then we have $(S,T)\models \varphi$, where $\varphi$ does not have to be an atom. So, intuitively, *downwards closure* of satisfaction in the double team framework is equivalent to downwards closure of atomic satisfaction.

It turns out that we do not actually need Lemma 2 in full generality. The essential part of the Lemma is that functions $f:U\rightarrow {\exists ^s}^{\mathfrak {A}}$ can be assumed to be $z$-independent; see the proof of Lemma 4 for further details.

Let $\psi$ be a sentence of $\hbox {DC}^2$. Define ${Dom}_{\psi }(\psi ) = \emptyset$. Assume then that we have defined ${Dom}_{\psi }(\chi )$ for $\chi \in \hbox {SUB}_{\psi }$.

1. If $\chi = \exists ^{\ge k} x\, \alpha$ or $\chi = \exists ^{s} x\, \alpha$, define ${Dom}_{\psi }(\alpha ) = {Dom}_{\psi }(\chi )\cup \{x\}$.
2. If $\chi = \exists ^{\ge k} y\, \alpha$ or $\chi = \exists ^{s} y\, \alpha$, define ${Dom}_{\psi }(\alpha ) = {Dom}_{\psi }(\chi )\cup \{y\}$.
3. If $$\chi = \chi _1\vee \chi _2$$, define $${Dom}_{\psi }(\chi _1) = {Dom}_{\psi }(\chi _2) = {Dom}_{\psi }(\chi )$$.
4. If $$\chi = \lnot \alpha $$, define $${Dom}_{\psi }(\alpha )= {Dom}_{\psi }(\chi )$$.

#### **Lemma 3**

Let $\psi$ be a sentence of  $\hbox {DC}^2$ and *U* a team with exactly one assignment. Then $\mathfrak {A},\bigl (U,\emptyset \bigr )\models \psi$ iff $\mathfrak {A},\bigl (\{\emptyset \},\emptyset \, \bigr )\models \psi$.

#### *Proof*

Let *s* be the unique assignment in *U*. Assume that $\mathfrak {A},(U,\emptyset )\models \psi$. The player $\fancyscript{A}$ has a uniform survival strategy *F* in the game $G\bigl (\{s\},\emptyset ,\psi \bigr )$. (Recall that we may write $G\bigl (U,\emptyset ,\psi \bigr )$ instead of $G\bigl (\mathfrak {A},U,\emptyset ,\psi \bigr )$.)

Now, let $F'$ be the strategy for $G(\{\emptyset \},\emptyset ,\psi )$, where $\fancyscript{A}$ canonically copies the moves determined by *F* in $G(\{ s\},\emptyset ,\psi )$. This means that for each position $(\mathfrak {A},t,\#, \alpha )$ in $G(\{\emptyset \},\emptyset ,\psi )$, we define $F'(\mathfrak {A},t,\#, \alpha ) := F(\mathfrak {A}, t',\#, \alpha )$, where $t = t' \upharpoonright {Dom}_{\psi }(\alpha )$, i.e., *t* is the restriction of $t'$ to the set ${Dom}_{\psi }(\alpha )$. It is easy to show that $F'$ is well-defined. Let $\chi$ be an arbitrary atom of $\psi$, and let $S(\chi )$ and $T(\chi )$ be the sets of positive and negative final assignments for $\chi$ in the game $G\bigl (\{s\},\emptyset ,\psi \bigr )$ when $\fancyscript{A}$ follows the strategy *F*. Recalling that $\psi$ is a sentence, it is easy to see that the teams of positive and negative final assignments $S^{*}(\chi )$ and $T^{*}(\chi )$ that arise in $G(\{\emptyset \},\emptyset ,\psi )$ when $\fancyscript{A}$ follows $F'$ are *essentially* the same teams as those that arise in $G(\{ s\},\emptyset ,\psi )$ when $\fancyscript{A}$ follows *F*: if $\chi$ has the tuple $\overline{z}$ of variables, we have ${Rel}\bigl (S^{*}(\chi ),\overline{z}\bigr ) = {Rel}\bigl (S^{}(\chi ),\overline{z}\bigr )$ and ${Rel}\bigl (T^{*}(\chi ),\overline{z}\bigr ) = {Rel}\bigl (T^{}(\chi ),\overline{z}\bigr )$. Therefore $\mathfrak {A},(\{\emptyset \},\emptyset \, )\models \psi$.

The converse implication is rather similar. Assume that $\mathfrak {A},(\{\emptyset \},\emptyset )\models \psi$. Thus $\fancyscript{A}$ has a uniform survival strategy $H$ in the game $G(\{\emptyset \},\emptyset ,\psi )$. Let $H'$ be the strategy for $G(\{\, s \},\emptyset ,\psi )$, where $\fancyscript{A}$ canonically copies the moves determined by $H$ in $G(\{ \emptyset \},\emptyset ,\psi )$. This means that for each position $(\mathfrak {A},t,\#, \alpha )$ in $G(\{\, s \},\emptyset ,\psi )$, we define $H'(\mathfrak {A},t,\#, \alpha ) := H(\mathfrak {A}, t',\#, \alpha )$, where $t' = t\upharpoonright {Dom}_{\psi }(\alpha )$. Let $\chi$ be an arbitrary atom of $\psi$, and let $S(\chi )$ and $T(\chi )$ be the sets of positive and negative final assignments for $\chi$ in the game $G\bigl (\{\emptyset \},\emptyset ,\psi \bigr )$ when $\fancyscript{A}$ follows the strategy $H$. It is easy to see that the teams of positive and negative final assignments $S^{*}(\chi )$ and $T^{*}(\chi )$ that arise in $G(\{ s \},\emptyset ,\psi )$ when $\fancyscript{A}$ follows $H'$ are *essentially* the same teams as those that arise in $G(\{ \emptyset \},\emptyset ,\psi )$ when $\fancyscript{A}$ follows *H*: if $\chi$ has the tuple $\overline{z}$ of variables, we have ${Rel}\bigl (S^{*}(\chi ),\overline{z}\bigr ) = {Rel}\bigl (S^{}(\chi ),\overline{z}\bigr )$ and ${Rel}\bigl (T^{*}(\chi ),\overline{z}\bigr ) = {Rel}\bigl (T^{}(\chi ),\overline{z}\bigr )$. Thus $\mathfrak {A},(\{ s \},\emptyset \, )\models \psi$.$\square$

Now *fix* a sentence $\varphi$ of $\hbox {DC}^2$. Our next aim is to define the $\hbox {FOC}^2$-sentence

$$\begin{aligned} \varphi ^*\ :=\ \psi _{initial}\ \wedge \ \bigwedge \limits _{\chi \ \in \ \hbox {SUB}_{\varphi }}\psi _{\chi } \end{aligned}$$

in full detail, and then prove that $\varphi$ and $\varphi ^*$ are equisatisfiable.

Let $\psi$ be an arbitrary subformula of $\varphi$. Having fixed the sentence $\varphi$, we shall write ${Dom}(\psi )$ instead of ${Dom}_{\varphi }(\psi )$ in the rest of the article. Let $\sigma$ be the set of relation symbols that occur in $\varphi$. As discussed above, $\varphi ^*$ contains extra relation symbols that encode information concerning subformulae of $\varphi$. Let $\hbox {QSUB}_{\varphi }$ denote the set of formulae $\alpha \in \hbox {SUB}_{\varphi }$ such that there exists another subformula $\psi = Qz\,\alpha \in \hbox {SUB}_{\varphi }$, where $Q\in \{\exists ^{\ge k}, \exists ^s\}$. For each formula $\alpha \in \hbox {QSUB}_{\varphi }$, define the fresh relation symbols $E_{\alpha }^{{ Uf}}$ and $E_{\alpha }^{Vg'}$. The arity of each of these symbols is $|{Dom}(\alpha )|$, i.e., the number of variables in ${Dom}(\alpha )$.

Additionally, for each formula $\chi \in \hbox {SUB}_{\varphi }$, define fresh relation symbols $S_{\chi }$ and $T_{\chi }$. The arity of the symbols $S_{\chi }$ and $T_{\chi }$ is equal to $max\{|{Dom}(\chi )|, 1\}$. The set of relation symbols in $\varphi ^*$ is the set

$$\begin{aligned}&\sigma \ \cup \ \{\, E_{\alpha }^{{ Uf}}\ |\ \alpha \, \in \, \hbox {QSUB}_{\varphi }\, \}\ \cup \ \{\, E_{\alpha }^{Vg'}\ |\ \alpha \, \in \, \hbox {QSUB}_{\varphi }\, \}\\&\quad \cup \ \{\, S_{\chi }\ |\ \chi \in \hbox {SUB}_{\varphi }\ \}\ \cup \ \{\, T_{\chi }\ |\ \chi \in \hbox {SUB}_{\varphi }\ \}. \end{aligned}$$

Let $\sigma ^*$ denote this set.

Define $\psi _{initial}\, :=\, \exists ^{=1} x\, S_{\varphi }(x)\wedge \lnot \exists x T_{\chi }(x)$. Here $\exists ^{=1}x$ is the $\hbox {FOC}^2$-expressible quantifier stating that exactly one *x* satisfies the quantified formula. To fully define $\varphi ^*$, we still need to define the formula $\psi _{\chi }$ for each formula $\chi \in \hbox {SUB}_{\varphi }$.

Let $\chi \in \hbox {SUB}_{\varphi }$. If $\chi = \chi _1\vee \chi _2$ and ${Dom}(\chi ) = \{x,y\}$, then $\psi _{\chi }$ is the conjunction of the formulae

$$\begin{aligned}&\psi _{\chi }^1\ := \forall x\forall y \Bigl (\, S_{\chi }(x,y)\ \leftrightarrow \ \bigl (S_{\chi _1}(x,y)\, \vee \, S_{\chi _2}(x,y)\bigr )\Bigr ),\\&\psi _{\chi }^2\ := \forall x\forall y \Bigl (\, T_{\chi }(x,y)\ \leftrightarrow \ T_{\chi _1}(x,y)\Bigr ),\\&\psi _{\chi }^3\ := \forall x\forall y \Bigl (\, T_{\chi }(x,y)\ \leftrightarrow \ T_{\chi _2}(x,y)\Bigr ). \end{aligned}$$

If $\chi = \exists ^{\ge k}y\, \alpha$ and ${Dom}(\chi ) = \{x,y\}$, then $\psi _{\chi }$ is the conjunction of the formulae

$$\begin{aligned}&\psi _{\chi }^1\ := \ \forall x\forall y\bigl (\ S_{\chi }(x,y)\ \rightarrow \ \exists ^{\ge k} y\, E_{\alpha }^{{ Uf}}(x,y)\, \bigr ),\\&\psi _{\chi }^2\ := \ \forall x\forall y\bigl (\, E_{\alpha }^{{ Uf}}(x,y)\ \rightarrow \ \exists y\, S_{\chi }(x,y)\, \bigr ),\\&\psi _{\chi }^3\ := \ \forall x\forall y\bigl (\ T_{\chi }(x,y)\ \rightarrow \ \lnot \exists ^{\ge k} y\, \lnot E_{\alpha }^{{ Vg}'}(x,y)\, \bigr ),\\&\psi _{\chi }^4\ := \ \forall x\forall y\bigl (\, E_{\alpha }^{{ Vg}'}(x,y)\ \rightarrow \ \exists y\, T_{\chi }(x,y)\, \bigr ),\\&\psi _{\chi }^5\ := \forall x \forall y\bigl ( S_{\alpha }(x,y)\ \leftrightarrow \ E_{\alpha }^{{ Uf}}(x,y)\, \bigr ),\\&\psi _{\chi }^6\ := \forall x \forall y\bigl (\, T_{\alpha }(x,y)\ \leftrightarrow \ E_{\alpha }^{{ Vg}'}(x,y)\, \bigr ). \end{aligned}$$

If $\chi$ is the atomic formula $=\!\!(x,y)$, and thus necessarily ${Dom}(\chi ) = \{x,y\}$, then $\psi _{\chi }$ is the conjunction of the formulae

$$\begin{aligned}&\psi _{\chi }^1\ :=\ \lnot \exists x\exists ^{\ge 2}y\, S_{\chi }(x,y),\\&\psi _{\chi }^2\ :=\ \lnot \exists x\exists y\, T_{\chi }(x,y). \end{aligned}$$

The structure of each formula $\psi _{\chi }$, where $\chi \in \hbox {SUB}_{\varphi }$, depends on $\chi$ and ${Dom}(\chi )$. A complete list of these formulae is given in the Appendix.

#### **Lemma 4**

Assume $\mathfrak {A}$ is a $\sigma$-model such that $\mathfrak {A},\bigl (\{\emptyset \},\emptyset \, \bigr )\models \varphi$. Let *A* be the domain of  $\mathfrak {A}$. Then there exists a $\sigma ^*$-model $\mathfrak {A}^*$ with the same domain *A* such that $\mathfrak {A}^*\models _{\mathrm{FO}}\varphi ^*$.

#### *Proof*

The relation symbols $R\in \sigma$ are interpreted in $\mathfrak {A}^*$ such that $R^{\mathfrak {A}^*} := R^{\mathfrak {A}}$. The interpretations of the relation symbols in $\sigma ^*\setminus \sigma$ are given below.

Let *U* is an team with domain $\{x\}$ and for the model $\mathfrak {A}$. Assume *U* contains exactly one assignment. Since $\mathfrak {A},(\{\emptyset \},\emptyset )\models \psi$, we have $\mathfrak {A},(U,\emptyset )\models \varphi$ by Lemma 3. We shall next recursively define a double team $(U_{\chi },V_{\chi })$ for each subformula $\chi \in \hbox {SUB}_\varphi$ such that $\mathfrak {A}, (U_{\chi },V_{\chi })\models \chi$ holds.

We shall simultaneously define the interpretations of the symbols in $\sigma ^*\setminus \sigma$, thereby completing the definition of the model $\mathfrak {A}^*$.

First define $(U_{\varphi },V_{\varphi }) := (U,\emptyset )$. Define $S_{\varphi }^{\mathfrak {A}^*} = {Rel}(U_{\varphi })$. Also define $T_{\varphi }^{\mathfrak {A}^*} :=\emptyset$. Now consider a formula $\chi \in \hbox {SUB}_{\varphi }$, and assume that we have defined $U_{\chi }$ and $V_{\chi }$ such that $\mathfrak {A},(U_{\chi },V_{\chi })\models \chi$.

Assume first that $\chi \, =\, \exists ^{\ge k} x\, \alpha$. As $\mathfrak {A},(U_{\chi },V_{\chi })\models \exists ^{\ge k} x\, \alpha$, there exist functions $f: U_{\chi }\rightarrow {\exists ^{\ge k}}^{\, \mathfrak {A}}$ and $g:V_{\chi }\rightarrow {\overline{\exists ^{\ge k}}}^{\, \mathfrak {A}}$ such that

$$\begin{aligned} \bigl (\, U_{\chi }[\, x/f\, ]\cup V_{\chi }[\, x/g\, ],\ U_{\chi }[\, x/f'\, ] \cup V_{\chi }[\, x/g'\, ]\, \bigr )\models \, \alpha . \end{aligned}$$

Furthermore, by Lemma 2, we assume, w.l.o.g., that the functions *f* and *g* are *x*-independent. We make the following definitions.

1. $$U_{\alpha }\, :=\, U_{\chi }[\, x/f\, ]\, \cup \, V_{\chi }[\, x/g\, ]=U_{\chi }[\, x/f\, ]$$
2. $$V_{\alpha }\, :=\, U_{\chi }[\, x/f'\, ]\, \cup \, V_{\chi }[\, x/g'\, ]\, =\, V_{\chi }[\, x/g'\, ]$$
3. $$S_{\alpha }^{\mathfrak {A}^*} := {Rel}\bigl (U_{\alpha }\bigr )$$
4. $$T_{\alpha }^{\mathfrak {A}^*} := {Rel}\bigl (V_{\alpha }\bigr )$$
5. $${E_{\alpha }^{{ Uf}}}^{\mathfrak {A}^*} := {Rel}\bigl (U_{\chi }[x/f]\bigr ) = S_{\alpha }^{\mathfrak {A}^*}$$
6. $${E_{\alpha }^{{ Vg}'}}^{\mathfrak {A}^*} := {Rel}\bigl (V_{\chi }[x/g']\bigr ) = T_{\alpha }^{\mathfrak {A}^*}$$

The cases where $\chi$ is a formula of any of the types $\exists ^{\ge k} y\, \alpha ,\ \exists ^{s} x\, \alpha ,\ \exists ^{s} y\, \alpha$, are treated analogously. It is essential—as we shall see—that the function *f* is *x*-independent in the case $\chi = \exists ^s x\, \alpha$ and *y*-independent when $\chi = \exists ^s y\, \alpha$.

Consider then the case where $\chi$ is $\alpha \vee \beta$. Since $(U_{\chi },V_{\chi })\models \alpha \vee \beta$, we have $(U_1,V_{\chi })\models \alpha$ and $(U_2,V_{\chi })\models \beta$ for some $U_1,U_2\subseteq U_{\chi }$ such that $U_1\cup U_2 = U_{\chi }$. We define $(U_{\alpha },V_{\alpha }) := (U_1, V_{\chi })$ and $(U_{\beta },V_{\beta }):= (U_2, V_{\chi })$. We also define $S_{\alpha }^{\mathfrak {A}^*} := {Rel}(U_{\alpha })$, $T_{\alpha }^{\mathfrak {A}^*} := {Rel}(V_{\alpha })$, $S_{\beta }^{\mathfrak {A}^*} := {Rel}(U_{\beta })$, and $T_{\beta }^{\mathfrak {A}^*} := {Rel}(V_{\beta })$.

In the case where $\chi$ is $\lnot \alpha$, we define $U_{\alpha } := V_{\chi }$ and $V_{\alpha } := U_{\chi }$. We also define $S_{\alpha }^{\mathfrak {A}^*} := {Rel}(U_{\alpha })$ and $T_{\alpha }^{\mathfrak {A}^*} := {Rel}(V_{\alpha })$.

We have now defined the teams $U_{\chi }$ and $V_{\chi }$ for each $\chi \in \hbox {SUB}_{\varphi }$ such that we have $\mathfrak {A},(U_{\chi },V_{\chi })\models \chi$. We have also fully defined a $\sigma ^*$-model $\mathfrak {A}^*$. We shall next show that $\mathfrak {A}^*\models _{\mathrm{FO}}\varphi ^*$. While it is clear that $\mathfrak {A}^*\models _{\mathrm{FO}}\psi _{initial}$, we must show that $\mathfrak {A}^*\models _{\mathrm{FO}} \psi _{\chi }$ for each $\chi \in \hbox {SUB}_{\varphi }$.

Let us first consider the case where $\chi$ is of the form $\exists ^{s}y\, \alpha$ for some $\alpha \in \hbox {SUB}_\varphi$. This case divides into further subcases, depending on ${Dom}(\chi )$.

We assume first that ${Dom}(\chi ) = \{x,y\}$.

We know that there exist *y*-independent functions $f:U_{\chi }\rightarrow {\exists ^{s}}^{\, \mathfrak {A}}$ and $g:V_{\chi }\rightarrow \overline{\exists ^{s}}^{\, \mathfrak {A}}$ such that

$$\begin{aligned} \mathfrak {A},\bigl (\, U_{\chi }[\, y/f\, ]\cup V_{\chi }[\, y/g\, ],\ U_{\chi }[\, y/f\, '\, ] \cup V_{\chi }[\, y/g\, '\, ]\, \bigr )\models \, \alpha . \end{aligned}$$

We have ${Rel}(U_{\chi }[\, y/f\, ]) = {E_{\alpha }^{{ Uf}}}^{\, \mathfrak {A}^*},\, {Rel}(V_{\chi }[\, y/g\, ]) = \emptyset ,\, {Rel}(U_{\chi }[\, y/f\,'\, ]) = \emptyset$ and ${Rel}(V_{\chi }[\, y/g\,'\, ]) = {E_{\alpha }^{{ Vg}'}}^{\, \mathfrak {A}^*}$.

We shall first show that $\mathfrak {A}^*\models _{\mathrm{FO}}\psi _{\chi }^1$. Here it is essential that the function *f* is *y*-independent. Assume that $\mathfrak {A}^*,[x\mapsto a,y\mapsto b]\models _{\mathrm{FO}} S_{\chi }(x,y)$. Thus $(a,b)\in S_{\chi }^{\mathfrak {A}^*} = {Rel}(U_{\chi })$. Since *f* is *y*-independent, there exists exactly one element $b'\in A$ such that $(a,b')\in {Rel}(U_{\chi }[ y/f]) = {E_{\alpha }^{{ Uf}}}^{\mathfrak {A}^*}$. Therefore we have $\mathfrak {A}^*,[x\mapsto a]\models _{\mathrm{FO}} \exists ^{=1}y\ {E_{\alpha }^{{ Uf}}}(x,y)$, as required.

We have $\mathfrak {A}^*\models _{\mathrm{FO}}\psi _{\chi }^2$ since for every assignment $s\in U_{\chi }[\, y/f ]$ such that $s(x) = a$, there must exist an assignment $s'\in U_{\chi }$ such that $s'(x) = a$. We can similarly show that $\mathfrak {A}^*\models _{\mathrm{FO}} \psi _{\chi }^3\wedge \psi _{\chi }^4$.

The fact that $\mathfrak {A}^*\models _{\mathrm{FO}} \psi _{\chi }^5\wedge \psi _{\chi }^6$ follows immediately since $U_{\alpha } = U_{\chi }[ y/f ]\, \cup \, V_{\chi }[ y/g ]$ and $V_{\alpha } = U_{\chi }[\, y/f\, '\, ]\, \cup \, V_{\chi }[\, y/g\, '\, ]$.

The cases where ${Dom}(\chi )$ is $\{x\}$, $\{y\}$, or $\emptyset$, are similar, as are the cases where $\chi := \exists ^{s}x\, \alpha$. Also all cases where $\chi := \exists ^{\ge k} y\, \alpha$ or $\exists ^{\ge k} x\, \alpha$ are similar; we shall discuss the details of the case where $\chi := \exists ^{\ge k} x\, \alpha$ and ${Dom}(\chi ) = \{x\}$.

We know that there exist functions $f:U_{\chi }\rightarrow {\exists ^{\ge k}}^{\mathfrak {A}}$ and $g:V_{\chi }\rightarrow {\overline{\exists ^{\ge k}}}^{\mathfrak {A}}$ such that

$$\begin{aligned} \mathfrak {A},\bigl (\, U_{\chi }[\, x/f\, ]\cup V_{\chi }[\, x/g\, ],\ U_{\chi }[\, x/f\, '\, ] \cup V_{\chi }[\, x/g\, '\, ]\, \bigr )\models \, \alpha . \end{aligned}$$

We have ${Rel}(U_{\chi }[\, x/f\, ]) = {E_{\alpha }^{{ Uf}}}^{\, \mathfrak {A}^*},\, {Rel}(V_{\chi }[\, x/g\, ]) = \emptyset ,\, {Rel}(U_{\chi }[\, x/f\,'\, ]) = \emptyset$ and ${Rel}(V_{\chi }[\, x/g\,'\, ]) = {E_{\alpha }^{{ Vg}'}}^{\, \mathfrak {A}^*}$. Let us show that $\mathfrak {A}^*\models _{\mathrm{FO}}\psi _{\chi }^1$. Assume that $\mathfrak {A}^*,[x\mapsto a]\models _{\mathrm{FO}} S_{\chi }(x)$ for some $a\in A$. Thus $S_{\chi }^{\mathfrak {A}^*} = {Rel}(U_{\chi }) \not = \emptyset$, whence $U_{\chi }\not =\emptyset$. Therefore there exist at least *k* elements $b\in A$ such that $b\in {Rel}(U_{\chi }[ x/f ]) = {E_{\alpha }^{{ Uf}}}^{\mathfrak {A}^*}$. Therefore $\mathfrak {A}^*\models _{\mathrm{FO}} \exists ^{\ge k}x\, {E_{\alpha }^{{ Uf}}}(x)$, as required.

We have $\mathfrak {A}^*\models _{\mathrm{FO}}\psi _{\chi }^2$ since if $U_{\chi }[\, x/f\, ] \not = \emptyset$, then $U_{\chi }\not =\emptyset$. To show that $\mathfrak {A}^*\models _{\mathrm{FO}}\psi _{\chi }^3$, assume that $\mathfrak {A}^*,[x\mapsto a]\models _{\mathrm{FO}} T_{\chi }(x)$ for some $a\in A$. Thus $T_{\chi }^{\mathfrak {A}^*} = {Rel}(V_{\chi })$ is not empty. Let $s\in V_{\chi }$. Recall that $g_2$ denotes the second coordinate function of *g*. By the definition of the minor quantifier $\exists ^{\ge k}$, there are at most $k-1$ elements in the set $A \setminus g_2(s)$. Thus there are at most $k-1$ elements in $A\setminus {Rel}\bigl (V_{\chi }[ x/g\, '\, ]\bigr )$. Therefore we have $\mathfrak {A}^*\models _{\mathrm{FO}} \lnot \exists ^{\ge k} x \lnot {E_{\alpha }^{{ Vg}'}}(x)$, and hence $\mathfrak {A}^*\models _{\mathrm{FO}}\psi _{\chi }^3$.

We have $\mathfrak {A}^*\models _{\mathrm{FO}} \psi _4$ since if $V_{\chi }[\, x/g\, '\, ]$ is not empty, then $V_{\chi }$ cannot be empty. We have $\mathfrak {A}^*\models _{\mathrm{FO}}\psi _5\wedge \psi _6$ since $U_{\alpha } = U_{\chi }[ x/f ]$ and $V_{\alpha } = V_{\chi }[\, x/g\, '\, ]$.

The cases where $\chi = \chi _1\vee \chi _2$ and $\chi = \lnot \alpha$ are straightforward, so we omit them and move directly to the cases where $\chi$ is an atomic formula. Assume first that $\chi = R(y,x)$ for some relation symbol *R*. We must show that $\mathfrak {A}^*\models _{\mathrm{FO}} \forall x\forall y\bigl (S_{R(y,x)}(x,y)\rightarrow R(y,x)\bigr ).$ (Notice indeed the order of all tuples of variables.) Assume that $\mathfrak {A}^*,[x\mapsto a, y\mapsto b]\models _{\mathrm{FO}} S_{R(y,x)}(x,y).$ By the definition of the relation $S_{R(y,x)}^{\mathfrak {A}^*}$, this means that $(a,b)\in {Rel}(U_{R(y,x)})$. We have $\mathfrak {A},(U_{R(y,x)},V_{R(y,x)})\models _{\mathrm{FO}}R(y,x)$, and therefore $\mathfrak {A}^*,s\models _{\mathrm{FO}} R(y,x)$ for all $s\in U_{R(y,x)}$. Thus $\mathfrak {A}^*,[x\mapsto a,y\mapsto b]\models _{\mathrm{FO}}R(y,x)$. All the remaining arguments for the cases where $\chi$ is an atomic first-order formula, are similar.

Assume then that $\chi$ is the atom $=\!\!(x,y)$. We must establish that we have $\mathfrak {A}^*\models _{\mathrm{FO}} \lnot \exists x\exists ^{\ge 2} y\ S_{=(x,y)}(x,y)$. Assume $\mathfrak {A}^*,[x\mapsto a,y\mapsto b]\models _{\mathrm{FO}} S_{=(x,y)}(x,y)$ for some $a,b\in A$. Therefore $(a,b)\in {Rel}(U_{=(x,y)})$. We have $\mathfrak {A},\bigl (U_{=(x,y)},V_{=(x,y)}\bigr )\models \ =\!\!(x,y),$ and therefore $s(y) = s'(y)$ for all $s,s'\in U_{=(x,y)}$ such that $s(x) = s'(x)$. Hence there exists no pair $(a,b')\in {Rel}(U_{=(x,y)}) = S_{=(x,y)}^{\mathfrak {A}^*}$ such that $b\not = b'$. Thus $\mathfrak {A}^*\models _{\mathrm{FO}} \lnot \exists x\exists ^{\ge 2} y\, S_{=(x,y)}(x,y)$, as required.

All remaining arguments concerning non-first-order atoms are similar.$\square$

#### **Lemma 5**

Let $\mathfrak {B}^*$ be a $\sigma ^*$-model such that $\mathfrak {B}^*\models _{\mathrm{FO}}\varphi ^*$. Let *B* be the domain of $\mathfrak {B}^*$. Then there exists a $\sigma$-model $\mathfrak {B}$ with the same domain *B* such that $\mathfrak {B},\bigl (\{\emptyset \},\emptyset \, \bigr )\models \varphi$.

#### *Proof*

Assume that $$\mathfrak {B}^*\models _{\mathrm{FO}}\varphi ^*$$.

Let $\mathfrak {B}$ be the reduct of $\mathfrak {B}^*$ to the vocabulary $\sigma$, i.e., the domain of $\mathfrak {B}$ is *B*, and each relation symbol $R\in \sigma$ is interpreted such that $R^{\mathfrak {B}} := R^{\mathfrak {B}^*}$.

We shall next define a double team $(U_{\chi },V_{\chi })$ for each $\chi \in \hbox {SUB}_{\varphi }$. We shall then establish that $\mathfrak {B},\bigl (U_{\chi },V_{\chi }\big )\models \chi$ for each $\chi \in \hbox {SUB}_{\varphi }$.

If ${Dom}(\chi )$ is any of the sets $\{x\}, \{y\}, \{x,y\}$, we let $U_{\chi }$ and $V_{\chi }$ be the teams with domain ${Dom}(\chi )$ and codomain *B* such that ${Rel}(U_{\chi }) = S_{\chi }^{\mathfrak {B}^*}$ and ${Rel}(V_{\chi }) = T_{\chi }^{\mathfrak {B}^*}$. If ${Dom}(\chi )$ is $\emptyset$, we let $U_{\chi }$ and $V_{\chi }$ be the teams with the domain $\{x\}$ and codomain *B* such that ${Rel}(U_{\chi }) = S_{\chi }^{\mathfrak {B}^*}$ and ${Rel}(V_{\chi }) = T_{\chi }^{\mathfrak {B}^*}$.

We shall prove by induction on the structure of $\varphi$ that $\mathfrak {B},\bigl (U_{\chi },V_{\chi }\bigr )\models \chi$ for each $\chi \in \hbox {SUB}_{\varphi }$. We shall then establish that $\mathfrak {B},\bigl (\{\emptyset \},\emptyset \, \bigr )\models \varphi$.

Assume first that $\chi$ is the atomic formula $R(y,x)$. Let $s\in U_{R(y,x)}$ be an assignment. Thus $\mathfrak {B}^*,s\models _{\mathrm{FO}} S_{R(y,x)}(x,y)$. Since $\mathfrak {B}^*\models _{\mathrm{FO}}\psi _{R(y,x)}$, we have $\mathfrak {B}^*,s\models _{\mathrm{FO}} R(y,x)$. We show similarly that if $t\in V_{R(y,x)}$, then $\mathfrak {B}^*,t\not \models R(y,x)$. Therefore $\mathfrak {B},\bigl (U_{R(y,x)},V_{R(y,x)}\bigr )\models R(y,x)$. The corresponding argument for other first-order atoms is similar.

Let $\chi$ be the atom $=\!\!(x,y)$. Since $\mathfrak {B}^*\models _{\mathrm{FO}}\psi _{\chi }$, there exist no pairs $(a,b),(a,b')\in S_{\chi }^{\mathfrak {B}^*}$ such that $b\not =b'$. Furthermore, $T_{\alpha }^{\mathfrak {B}^*} = \emptyset$. Therefore $\mathfrak {B},(U_{\chi },V_{\chi })\models \chi$. The corresponding arguments for other non-first-order atoms of $\hbox {DC}^2$ are similar.

For the sake of induction, let $\chi \, :=\, \exists ^{\ge k} y\, \alpha$ be a subformula of $\varphi$, and assume that $\mathfrak {B},(U_{\alpha },V_{\alpha })\models \alpha$. We need to show that $\mathfrak {B},(U_{\chi },V_{\chi })\models \chi$. Let us consider the case where ${Dom}(\chi ) = \{x,y\}$. We define a function $f:U_{\chi }\rightarrow {\exists ^{\ge k}}^{\, \mathfrak {B}}$ as follows. Assume $s\in U_{\chi }$ is an assignment such that $s(x) = a$ and $s(y) = b$ for some $a,b\in B$. Thus $(a,b)\in S_{\chi }^{\mathfrak {B}^*}$. Since $\mathfrak {B}^*\models _{\mathrm{FO}}\psi _{\chi }^1$, the set

4 $$\begin{aligned} B_s\ :=\ \{\, c\in A\ |\ \mathfrak {B}^*,[\, x\mapsto a,\, y\mapsto c\, ] \models E_{\alpha }^{{ Uf}}(x,y)\ \} \end{aligned}$$

has at least *k* elements. Define $f:U_{\chi }\rightarrow {\exists ^{\ge k}}^{\, \mathfrak {B}}$ such that $f(s) := (B_s,\emptyset )$ for each $s\in U_{\chi }$. Thus ${Rel}( U_{\chi }[\, y/f\, ]) \subseteq {E_{\alpha }^{{ Uf}}}^{\mathfrak {B}^*}$.

Let us then similarly define a function $g:V_{\chi }\rightarrow {\overline{\exists ^{\ge k}}}^{\, \mathfrak {B}}$. Let $s\in V_{\chi }$ be an assignment such that $s(x) = a$ and $s(y) = b$ for some $a,b\in B$. Thus $(a,b)\in T_{\chi }^{\mathfrak {B}^*}$. Since $\mathfrak {B}^*\models _{\mathrm{FO}}\psi _{\chi }^3$, the number of elements in the set

5 $$\begin{aligned} C_s\ :=\ \{\, c\in A\ |\ \mathfrak {B}^*,[\, x\mapsto a,\, y\mapsto c\, ]\models E_{\chi }^{{ Vg}'}(x,y)\ \} \end{aligned}$$

satisfies the condition $|B\setminus C_{s}| < k$. Define $g:V_{\chi }\rightarrow \overline{\exists ^{\ge k}}^{{}\, \mathfrak {B}}$ such that $g(s) := (\emptyset ,C_s)$ for each $s\in V_{\chi }$. Thus ${Rel}( V_{\chi }[\, y/g\, '\, ]) \subseteq {E_{\alpha }^{{ Vg}'}}^{\, \mathfrak {B}^*}$.

As $U_{\chi }[\, y/f\, '\, ] = V_{\chi }[\, y/g\, ] = \emptyset$, we now know that

6 $$\begin{aligned} {Rel}\bigl (\, U_{\chi }[\, y/f\, ]\, )\, \cup \, {Rel}(\, V_{\chi }[\, y/g\, ]\, \bigr )\ \subseteq \ {E_{\alpha }^{{ Uf}}}^{\mathfrak {B}^*} \end{aligned}$$

and

7 $$\begin{aligned} {Rel}\bigl (\, U_{\chi }[\, y/f\, ' \, ]\, )\, \cup \, {Rel}(\, V_{\chi }[\, y/g\, ' \, ]\, \bigr )\ \subseteq \ {E_{\alpha }^{{ Vg}'}}^{\mathfrak {B}^*}. \end{aligned}$$

We then show that also the converse inclusion of Eq. 6 holds. Assume that $(a,c)\in {E_{\alpha }^{{ Uf}}}^{\mathfrak {B}^*}$. As $\mathfrak {B}^*\models _{\mathrm{FO}}\psi _{\chi }^2$, there exists some $b\in B$ such that $(a,b)\in {Rel}(U_{\chi })$. Let $s\in U_{\chi }$ be the assignment such that $s(x) = a$ and $s(y) = b$. Now, by the definition of *f* (see Eq. 4), we observe that since $(a,c)\in {E_{\alpha }^{{ Uf}}}^{\mathfrak {B}^*}$, we have $c\in f_1(s)$; recall here that $f_1$ denotes the first coordinate function of *f*. Thus $(a,c)\in {Rel}(U_{\chi }[\, y/f\, ])$. Therefore the converse inclusion of Eq. 6 holds.

We then establish that also the converse inclusion of Eq. 7 holds. Assume that $(a,c)\in {E_{\alpha }^{{ Vg}'}}^{\mathfrak {B}^*}$. As $\mathfrak {B}^*\models _{\mathrm{FO}}\psi _{\chi }^4$, there exists some $b\in B$ such that $(a,b)\in {Rel}(V_{\chi })$. Let $s\in V_{\chi }$ be the assignment such that $s(x) = a$ and $s(y) = b$. By the definition of the function *g* (Eq. 5), we observe that $c\in g_2(s)$. Thus $(a,c)\in {Rel}(V_{\chi }[\, y/g\, ' \, ])$. Hence the converse inclusion of Eq. 7 holds.

As $\mathfrak {B}^*\models _{\mathrm{FO}}\psi _{\chi }^5\wedge \psi _{\chi }^6$, we conclude that $U_{\chi }[\, y/f\, ]\cup V_{\chi }[\, y/g\, ] = U_{\alpha }$ and $V_{\chi }[\, y/f\, ' \, ]\cup V_{\chi }[\, y/g\, ' \, ] = V_{\alpha }$. As $\mathfrak {B},(U_{\alpha },V_{\alpha })\models \alpha$, we therefore conclude that $\mathfrak {B}, (U_{\chi },V_{\chi })\models \chi$. The remaining cases where $\chi = \exists ^{\ge k} y\, \alpha$ or $\,\chi = \exists ^{\ge k} x\, \alpha$, are similar. We next deal with the strict existential quantifier $\exists ^s$.

Let $\chi \, :=\, \exists ^{s} x\, \alpha$, and assume $\mathfrak {B},(U_{\alpha },V_{\alpha })\models \alpha$. Let us consider the details of the case where ${Dom}(\chi ) = \{y\}$. We define a function $f:U_{\chi }\rightarrow {\exists ^{s}}^{\, \mathfrak {B}}$ as follows. Assume $s\in U_{\chi }$ is an assignment such that $s(y) = a$ for some $a\in B$. Thus $a\in S_{\chi }^{\mathfrak {B}^*}$. Since $\mathfrak {B}^*\models _{\mathrm{FO}}\psi _{\chi }^1$, the size of the set

8 $$\begin{aligned} B_s\ :=\ \{\, c\in A\ |\ \mathfrak {B}^*,[\, x\mapsto c,\, y\mapsto a\, ] \models E_{\alpha }^{{ Uf}}(x,y)\, \} \end{aligned}$$

is exactly one. Define $f:U_{\chi }\rightarrow {\exists ^{s}}^{\, \mathfrak {B}}$ such that $f(s) := (B_s,\emptyset )$ for each $s\in U_{\chi }$. Thus ${Rel}( U_{\chi }[\, x/f\, ]) \subseteq {E_{\alpha }^{{ Uf}}}^{\mathfrak {B}^*}$. We also of course have ${Rel}( U_{\chi }[\, x/f\, '\, ]) = \emptyset$.

Let us then define the function $g:V_{\chi }\rightarrow {\overline{\exists ^{s}}}^{\, \mathfrak {B}}$ such that $g(s) = (\emptyset ,B)$ for each $s\in V_{\chi }$. Assume $s\in V_{\chi }$ is an assignment such that $s(y) = a$. Thus $a\in T_{\chi }^{\mathfrak {B}^*}$. Since $\mathfrak {B}^*\models _{\mathrm{FO}}\psi _{\chi }^3$, we have $(c,a) \in {E_{\alpha }^{{ Vg}'}}^{\, \mathfrak {B}^*}$ for each $c\in A$. Thus ${Rel}( V_{\chi }[\, x/g\, '\, ]) \subseteq {E_{\alpha }^{{ Vg}'}}^{\, \mathfrak {B}^*}$.

As ${Rel}( V_{\chi }[\, x/g\, ])$ and ${Rel}( V_{\chi }[\, x/f\, ' \, ])$ are empty, we have

9 $$\begin{aligned} {Rel}\bigl (\, U_{\chi }[\, x/f\, ]\, )\, \cup \, {Rel}(\, V_{\chi }[\, x/g\, ]\, \bigr )\ \subseteq \ {E_{\alpha }^{{ Uf}^{\mathfrak {B}}{^{*}}}} \end{aligned}$$

and

10 $$\begin{aligned} {Rel}\bigl (\, U_{\chi }[\, x/f\, ' \, ]\, )\, \cup \, {Rel}(\, V_{\chi }[\, x/g\, ' \, ]\, \bigr )\ \subseteq \ {E_{\alpha }^{{ Vg}'}}^{\mathfrak {B}^*}. \end{aligned}$$

We then show that the converse inclusion of Eq. 9 holds. Assume that $(a,b)\in {E_{\alpha }^{{ Uf}}}^{\mathfrak {B}^*}$. As $\mathfrak {B}^*\models _{\mathrm{FO}}\psi _{\chi }^2$, we have $b\in {Rel}(U_{\chi })$. Let $s\in U_{\chi }$ be the assignment such that $s(y) = b$. Now, by the definition of *f* (see Eq. 8), since $(a,b)\in {E_{\alpha }^{{ Uf}}}^{\mathfrak {B}^*}$, we have $(a,b)\in {Rel}\bigl (U[\, x/f\, ]\bigr )$. Therefore the converse inclusion of Eq. 9 holds.

It is easy to establish that also the converse inclusion of Eq. 10 holds. Therefore, as $\mathfrak {B}^*\models _{\mathrm{FO}} \psi _{\chi }^5\wedge \psi _{\chi }^6$, we infer that $U_{\chi }[\, y/f\, ]\cup V_{\chi }[\, y/g\, ] = U_{\alpha }$ and $V_{\chi }[\, y/f\, ' \, ]\cup V_{\chi }[\, y/g\, ' \, ] = V_{\alpha }$. As $\mathfrak {B},(U_{\alpha },V_{\alpha })\models \alpha$, we therefore conclude that $\mathfrak {B}, (U_{\chi },V_{\chi })\models \chi$.

We have now discussed the cases where $\chi = \exists ^{\ge k} z\, \alpha$ or $\chi = \exists ^{s} z\, \alpha$; here $z\in \{x,y\}$. The arguments for the cases where $\chi = \alpha \vee \beta$ or $\chi = \lnot \alpha$, are straightforward.

We conclude that $\mathfrak {B},(U_{\varphi },V_{\varphi })\models \varphi$. Since $\mathfrak {B}^*\models \psi _{initial}$, we have ${Rel}(U_{\varphi }) = S_{\varphi }^{\mathfrak {B}^*} = \{b\}$ for some $b\in B$ and ${Rel}(V_{\varphi }) = T_{\varphi }^{\mathfrak {B}^*} =\emptyset$. Hence $\mathfrak {B},\bigl (\{\emptyset \},\emptyset \, \bigr )\models \varphi$ by Lemma 3. $\square$

#### **Theorem 2**

The satisfiability and finite satisfiability problems of  $\hbox {DC}^2$ are complete for $\hbox {NEXPTIME}$.

#### *Proof*

The satisfiability and finite satisfiability problems of $\hbox {DC}^2$ are in $\hbox {NEXPTIME}$ due to the translation from $\hbox {DC}^2$ into $\hbox {FOC}^2$ defined above; it is shown in Pratt-Hartmann (2005) that the satisfiability and finite satisfiability problems for $\hbox {FOC}^2$ are $\hbox {NEXPTIME}$-complete. Furthermore, the satisfiability and finite satisfiability problems for $\hbox {DC}^2$ are $\hbox {NEXPTIME}$-hard since $\hbox {DC}^2$ contains $\hbox {FOC}^2$.$\square$

By analyzing our proofs above, we notice that for example the total existential quantifier $\exists ^t$ could be added to $\hbox {DC}^2$ without sacrificing $\hbox {NEXPTIME}$-completeness. The related formulae for the case where $\chi = \exists ^t y\, \alpha$ and ${Dom}(\chi ) = \{x,y\}$, are listed below. We let $\tilde{\vee }$ denote the *exclusive disjunction* and use a novel symbol $E_{\alpha }^{{\textit{Uf}}'}$ that corresponds to the team $U_{\chi }[\, y/f\, '\, ]$.

$$\begin{aligned}&\psi _{\chi }^1\ := \ \forall x\forall y\bigl (\, S_{\chi }(x,y)\ \rightarrow \ \exists y\, E_{\alpha }^{{ Uf}}(x,y)\, \bigr ),\\&\psi _{\chi }^2\ :=\ \forall x\forall y\Bigl (\, S_{\chi }(x,y) \rightarrow \forall y \bigl ( E_{\alpha }^{{ Uf}}(x,y)\, \tilde{\vee }\, E_{\alpha }^{{ {Uf}}'}(x,y)\bigr )\Bigr ),\\&\psi _{\chi }^3\ := \ \forall x\forall y\Bigl (\, \bigl (E_{\alpha }^{{ Uf}}(x,y)\vee E_{\alpha }^{{ {Uf}}'}(x,y)\bigr )\ \rightarrow \ \exists y\, S_{\chi }(x,y)\, \Bigr ),\\&\psi _{\chi }^4\ := \ \forall x\forall y\bigl (\ T_{\chi }(x,y)\ \rightarrow \ \forall y\, E_{\alpha }^{{ Vg}'}(x,y)\, \bigr ),\\&\psi _{\chi }^5\ := \ \forall x\forall y\Bigl (\, E_{\alpha }^{{ Vg}'}(x,y)\ \rightarrow \ \exists y\, T_{\chi }(x,y)\, \Bigr ),\\&\psi _{\chi }^7\ :=\ \forall x \forall y\Bigl ( S_{\alpha }(x,y)\ \leftrightarrow \ E_{\alpha }^{{ Uf}}(x,y)\, \Bigr ),\\&\psi _{\chi }^8\ :=\ \forall x \forall y\Bigl ( T_{\alpha }(x,y)\ \leftrightarrow \ \bigl (E_{\alpha }^{{ {Uf}}'}(x,y)\vee E_{\alpha }^{{ Vg}'}(x,y)\bigr )\Bigr ). \end{aligned}$$

Interestingly, it is essential for our argument here that *f* can be assumed to be *y*-independent. Otherwise we would run into trouble with the formula $\psi _{\chi }^2$.

## A Semantics for Single Teams

In this section we define a semantics for variants of dependence logic with generalized quantifiers based on single teams. We also modify the notion of a generalized atom so that it works naturally in this context. Let us first define the following semantics with two turnstiles $\models ^+$ and $\models ^-$.

$$\begin{aligned} \begin{array}{lll} \mathfrak {A},U\models ^+ y_1=y_2 &{} \ \Leftrightarrow \ &{} \forall s\in U\bigl (\mathfrak {A},s\models _{\mathrm{FO}} y_1=y_2\bigr ).\\ \mathfrak {A},U\models ^- y_1=y_2 &{} \ \Leftrightarrow \ &{} \forall s\in U\bigl (\mathfrak {A},s\models _{\mathrm{FO}} y_1\not =y_2\bigr ).\\ \mathfrak {A},U\models ^+ R(y_1,\ldots ,y_m)&{} \ \Leftrightarrow \ &{} \forall s\in U\bigl (\mathfrak {A},s\models _{\mathrm{FO}} R(y_1,\ldots ,y_m)\bigr ).\\ \mathfrak {A},U\models ^- R(y_1,\ldots ,y_m)&{} \ \Leftrightarrow \ &{} \forall s\in U\bigl (\mathfrak {A},s\not \models _{\mathrm{FO}} R(y_1,\ldots ,y_m)\bigr ).\\ \mathfrak {A},U\models ^+ \lnot \varphi &{} \ \Leftrightarrow \ &{} \mathfrak {A},U\models ^- \varphi .\\ \mathfrak {A},U\models ^- \lnot \varphi &{} \ \Leftrightarrow \ &{} \mathfrak {A},U\models ^+ \varphi .\\ \mathfrak {A},U\models ^+(\varphi \vee \psi ) &{} \ \Leftrightarrow \ &{} \mathfrak {A},U_1\models ^+\varphi \hbox { and } \mathfrak {A},U_2\models ^+\psi \hbox { for}\\ &{} &{}\hbox {some }U_1,U_2\subseteq U\hbox { such that } U_1\cup U_2 = U.\\ \mathfrak {A},U\models ^-(\varphi \vee \psi ) &{} \ \Leftrightarrow \ &{} \mathfrak {A},U\models ^-\varphi \hbox { and } \mathfrak {A},U\models ^-\psi .\\ \end{array} \end{aligned}$$

For a generalized quantifier *Q* of type $(i_1,\ldots ,i_n)$, the semantic clause is defined such that $\mathfrak {A},U\models ^+ Q\overline{x}_1,\ldots ,\overline{x}_n(\varphi _1,\ldots ,\varphi _n)$ iff there exists a function $f:U\rightarrow Q^{\mathfrak {A}}$ such that

$$\begin{aligned} \begin{array}{c} \mathfrak {A},U[\, \overline{x}_1/f_1]\models ^+\varphi _1\hbox { and } \, U[\, \overline{x}_1/{f_1}'\, ]\models ^-\varphi _1,\\ \vdots \\ \mathfrak {A},U[\, \overline{x}_n/f_n]\models ^+\varphi _n\hbox { and } \, U[\, \overline{x}_n/{f_n}'\, ]\models ^-\varphi _n.\\ \end{array} \end{aligned}$$

We also define that $\mathfrak {A},U\models ^-Q\overline{x}_1,\ldots ,\overline{x}_n(\varphi _1,\ldots ,\varphi _n)$ iff there exists a function $g:U\rightarrow \overline{Q}^{\mathfrak {A}}$ such that

$$\begin{aligned} \begin{array}{c} \mathfrak {A},U[\, \overline{x}_1/g_1]\models ^+\varphi _1\hbox { and } \, U[\, \overline{x}_1/{g_1}'\, ]\models ^-\varphi _1,\\ \vdots \\ \mathfrak {A},U[\, \overline{x}_n/g_n]\models ^+\varphi _n\hbox { and } \, U[\, \overline{x}_n/{g_n}'\, ]\models ^-\varphi _n.\\ \end{array} \end{aligned}$$

It is straightforward to establish the following proposition.

### **Proposition 4**

Let $\varphi$ be a formula of first-order logic, possibly extended with generalized quantifiers. Let *U* be a team. Then the equivalences $\mathfrak {A},U\models ^+\varphi \ \Leftrightarrow \ \forall s\in U(\mathfrak {A},s\models _{\mathrm{FO}}\varphi )$ and $\mathfrak {A},U\models ^-\varphi \ \Leftrightarrow \ \forall s\in U(\mathfrak {A},s\not \models _{\mathrm{FO}}\varphi )$ hold.

For a minor quantifier *M*, we define $\mathfrak {A},U\models ^+ M x\, \varphi$ if and only if there exists a function $f:U\rightarrow M^{\mathfrak {A}}$ such that $\mathfrak {A},U[\, x/f]\models ^+\varphi \,\hbox {and} \, \mathfrak {A},U[\, x/f'\, ]\models ^-\varphi .$ We also define $\mathfrak {A},U\models ^-M x\, \varphi$ if and only if there exists a function $g:U\rightarrow \overline{M}^{\mathfrak {A}}$ such that $\mathfrak {A},U[\, x/g]\models ^+\varphi \,\hbox {and} \, \mathfrak {A},U[\, x/g'\, ]\models ^-\varphi .$ If *Q* is a generalized quantifier and $M\le Q$ its minor, we can replace any instance of *Q* by *M* or vice versa, without affecting the satisfaction of formulae. Note, however, that this interchangeability does not generally hold if we add generalized atoms into the picture.

Indeed, we can naturally extend the single team framework with a suitable notion of a generalized atom. Let $(Q,P)$ be a pair of generalized quantifiers, each of type $(i_1,\ldots ,i_k)$. Consider syntactic atomic expressions of type $A(\overline{y}_1,\ldots ,\overline{y}_k)$, where each $\overline{y}_j$ is of the length $i_j$. We define

$$\begin{aligned} \mathfrak {A},U\models ^+A(\overline{y}_1,\ldots ,\overline{y}_k)\ \Leftrightarrow \ \bigl ({Rel}(U,\mathfrak {A},\overline{y}_1),\ldots ,{Rel}(U,\mathfrak {A},\overline{y}_k)\bigr ) \in Q^{\mathfrak {A}} \end{aligned}$$

and

$$\begin{aligned} \mathfrak {A},U\models ^-A(\overline{y}_1,\ldots ,\overline{y}_k)\ \Leftrightarrow \ \bigl ({Rel}(U,\mathfrak {A},\overline{y}_1),\ldots ,{Rel}(U,\mathfrak {A},\overline{y}_k)\bigr ) \in P^{\mathfrak {A}}. \end{aligned}$$

It is not difficult to devise a corresponding symmetric game-theoretic semantics for single teams, but we shall not do this in the current article for the sake of brevity. The uniformity condition here seems to be—in a subtle way—quite different from the uniformity condition of the game theoretic semantics corresponding to the double team semantics. Indeed, we *do not* claim that the single team semantics is simply another way of presenting the double team semantics. The analysis of the differences between the single team and double team semantics is left for the future.

## Concluding Remarks

We have defined the notions of a generalized atom and minor quantifier, and shown how these notions can be used in defining extensions and variants of dependence logic. We have devised a double team semantics that can accommodate such extensions and variants under the same umbrella framework in a natural way. We have established that the double team semantics has a natural game-theoretic counterpart, and discussed issues related to the interpretation of logics based on team semantics. We have put double team semantics into use by defining the extension $\hbox {DC}^2$ of $\hbox {D}^2$ with counting quantifiers. We have shown that the satisfiability and finite satisfiability problems of $\hbox {DC}^2$ are complete for $\hbox {NEXPTIME}$.

Obvious interesting future questions involve the investigation of logics that mix different minor quantifiers and generalized atoms. It is also interesting to see *how natural* generalized atoms are in logical investigations. Phenomena that appear strange arise easily in logics that belong to the family of independence-friendly logic, often because technical operators are carelessly associated with intuitions that arise from the use of the same symbols in first-order logic. Signalling (see Mann et al. 2011) is a classical example of such a phenomenon. It remains to be investigated what kinds of systems embeddable in the double team semantics are natural, and up to what extent. For example the notion of negation calls for further analysis in this context.

There already exists a wide range of papers on logics based on team semantics. Subtle changes in semantic choices, such as using the lax existential quantifier instead of the strict one, lead to logics with different expressivities. To understand related phenomena better, it definitely makes sense to study systems based on team semantics in a unified framework. The double team semantics aims to provide such a framework.

## Appendix: Formulae for the Translation $\hbox {DC}^2\rightarrow \hbox {FOC}^2$

### Formulae for $\chi = \exists ^{\ge k} x\, \alpha$.

$${Dom}(\chi ) = \{x,y\}$$:

$$\begin{aligned}&\psi _{\chi }^1\ := \ \forall x\forall y\bigl (\ S_{\chi }(x,y)\ \rightarrow \ \exists ^{\ge k} x\, E_{\alpha }^{{ Uf}}(x,y)\, \bigr ),\\&\psi _{\chi }^2\ := \ \forall x\forall y\bigl (\, E_{\alpha }^{{ Uf}}(x,y)\ \rightarrow \ \exists x\, S_{\chi }(x,y)\, \bigr ),\\&\psi _{\chi }^3\ := \ \forall x\forall y\bigl (\ T_{\chi }(x,y)\ \rightarrow \ \lnot \exists ^{\ge k} x\, \lnot E_{\alpha }^{{ Vg}'}(x,y)\, \bigr ),\\&\psi _{\chi }^4\ := \ \forall x\forall y\bigl (\, E_{\alpha }^{{ Vg}'}(x,y)\ \rightarrow \ \exists x\, T_{\chi }(x,y)\, \bigr ),\\&\psi _{\chi }^5\ := \forall x \forall y\bigl ( S_{\alpha }(x,y)\ \leftrightarrow \ E_{\alpha }^{{ Uf}}(x,y)\bigr ),\\&\psi _{\chi }^6\ := \forall x \forall y\bigl (\, T_{\alpha }(x,y)\ \leftrightarrow \ E_{\alpha }^{{ Vg}'}(x,y)\, \bigr ). \end{aligned}$$

${Dom}(\chi )$ is either of the sets $\{x\},\ \emptyset$:

$$\begin{aligned}&\psi _{\chi }^1\ := \ \exists x\, S_{\chi }(x)\ \rightarrow \ \exists ^{\ge k} x\, E_{\alpha }^{{ Uf}}(x),\\&\psi _{\chi }^2\ := \ \exists x\, E_{\alpha }^{{ Uf}}(x)\ \rightarrow \ \exists x\, S_{\chi }(x),\\&\psi _{\chi }^3\ := \ \exists x\, T_{\chi }(x)\ \rightarrow \ \lnot \exists ^{\ge k} x\, \lnot E_{\alpha }^{{ Vg}'}(x),\\&\psi _{\chi }^4\ := \ \exists x E_{\alpha }^{{ Vg}'}(x)\ \rightarrow \ \exists x\, T_{\chi }(x),\\&\psi _{\chi }^5\ :=\ \forall x \bigl (\, S_{\alpha }(x)\ \leftrightarrow \ E_{\alpha }^{{ Uf}}(x)\, \bigr ),\\&\psi _{\chi }^6\ :=\ \forall x \bigl (\, T_{\alpha }(x)\ \leftrightarrow \ E_{\alpha }^{{ Vg}'}(x)\, \bigr ). \end{aligned}$$

$${Dom}(\chi )$$ is $$\{y\}$$:

$$\begin{aligned}&\psi _{\chi }^1\ := \ \forall y\bigl (\ S_{\chi }(y)\ \rightarrow \ \exists ^{\ge k} x\, E_{\alpha }^{{ Uf}}(x,y)\, \bigr ),\\&\psi _{\chi }^2\ := \ \forall x\forall y\bigl (\, E_{\alpha }^{{ Uf}}(x,y)\ \rightarrow \ S_{\chi }(y)\, \bigr ),\\&\psi _{\chi }^3\ := \ \forall y \bigl (\ T_{\chi }(y)\ \rightarrow \ \lnot \exists ^{\ge k} x\, \lnot E_{\alpha }^{{ Vg}'}(x,y)\, \bigr ),\\&\psi _{\chi }^4\ := \ \forall x\forall y\bigl (\, E_{\alpha }^{{ Vg}'}(x,y)\ \rightarrow \ T_{\chi }(y)\, \bigr ),\\&\psi _{\chi }^5\ := \forall x \forall y\bigl ( S_{\alpha }(x,y)\ \leftrightarrow \ E_{\alpha }^{{ Uf}}(x,y)\, \bigr ),\\&\psi _{\chi }^6\ := \forall x \forall y\bigl (\, T_{\alpha }(x,y)\ \leftrightarrow \ E_{\alpha }^{{ Vg}'}(x,y)\, \bigr ). \end{aligned}$$

### Formulae for $\chi = \exists ^{\ge k} y\, \alpha$.

$${Dom}(\chi ) = \{x,y\}$$:

$$\begin{aligned}&\psi _{\chi }^1\ := \ \forall x\forall y\bigl (\ S_{\chi }(x,y)\ \rightarrow \ \exists ^{\ge k} y\, E_{\alpha }^{{ Uf}}(x,y)\, \bigr ),\\&\psi _{\chi }^2\ := \ \forall x\forall y\bigl (\, E_{\alpha }^{{ Uf}}(x,y)\ \rightarrow \ \exists y\, S_{\chi }(x,y)\, \bigr ),\\&\psi _{\chi }^3\ := \ \forall x\forall y\bigl (\ T_{\chi }(x,y)\ \rightarrow \ \lnot \exists ^{\ge k} y\, \lnot E_{\alpha }^{{ Vg}'}(x,y)\, \bigr ),\\&\psi _{\chi }^4\ := \ \forall x\forall y\bigl (\, E_{\alpha }^{{ Vg}'}(x,y)\ \rightarrow \ \exists y\, T_{\chi }(x,y)\, \bigr ),\\&\psi _{\chi }^5\ := \forall x \forall y\bigl (\, S_{\alpha }(x,y)\ \leftrightarrow \ E_{\alpha }^{{ Uf}}(x,y)\, \bigr ),\\&\psi _{\chi }^6\ := \forall x \forall y\bigl (\, T_{\alpha }(x,y)\ \leftrightarrow \ E_{\alpha }^{{ Vg}'}(x,y)\, \bigr ). \end{aligned}$$

If ${Dom}(\chi )$ is either of the sets $\{y\}, \emptyset$, exactly the same formulae $\psi _{\chi }^1,\ldots ,\psi _{\chi }^6$ are used as in the case where $\chi = \exists ^{\ge k} x\, \alpha$ and ${Dom}(\chi )$ is $\{x\}$ or $\emptyset$.

$${Dom}(\chi )$$ is $$\{x\}$$:

$$\begin{aligned}&\psi _{\chi }^1\ := \ \forall x\bigl (\ S_{\chi }(x)\ \rightarrow \ \exists ^{\ge k} y\, E_{\alpha }^{{ Uf}}(x,y)\, \bigr ),\\&\psi _{\chi }^2\ := \ \forall x\forall y\bigl (\, E_{\alpha }^{{ Uf}}(x,y)\ \rightarrow \ S_{\chi }(x)\, \bigr ),\\&\psi _{\chi }^3\ := \ \forall x\bigl (\ T_{\chi }(x)\ \rightarrow \ \lnot \exists ^{\ge k} y\, \lnot E_{\alpha }^{{ Vg}'}(x,y)\, \bigr ),\\&\psi _{\chi }^4\ := \ \forall x\forall y\bigl (\ E_{\alpha }^{{ Vg}'}(x,y)\ \rightarrow \ T_{\chi }(x)\, \bigr ),\\&\psi _{\chi }^5\ := \forall x \forall y\bigl ( S_{\alpha }(x,y)\ \leftrightarrow \ E_{\alpha }^{{ Uf}}(x,y)\, \bigr ),\\&\psi _{\chi }^6\ := \forall x \forall y\bigl (\, T_{\alpha }(x,y)\ \leftrightarrow \ E_{\alpha }^{{ Vg}'}(x,y)\, \bigr ). \end{aligned}$$

### Formulae for $\chi = \exists ^{s} x\, \alpha$.

Below we let $\exists ^{= 1} x\ \psi$ denote the ($\hbox {FOC}^2$-expressible) condition that exactly one *x* satisfies $\psi$.

$${Dom}(\chi ) = \{x,y\}$$:

$$\begin{aligned}&\psi _{\chi }^1\ := \ \forall x\forall y\bigl (\ S_{\chi }(x,y)\ \rightarrow \ \exists ^{= 1} x\, E_{\alpha }^{{ Uf}}(x,y)\, \bigr ),\\&\psi _{\chi }^2\ := \ \forall x\forall y\bigl (\, E_{\alpha }^{{ Uf}}(x,y)\ \rightarrow \ \exists x\, S_{\chi }(x,y)\, \bigr ),\\&\psi _{\chi }^3\ :=\ \forall x\forall y\bigl (\, T_{\chi }(x,y) \rightarrow \forall x E_{\alpha }^{{ Vg}'}(x,y)\bigr ),\\&\psi _{\chi }^4\ := \ \forall x\forall y\bigl (\, E_{\alpha }^{{ Vg}'}(x,y)\ \rightarrow \ \exists x\, T_{\chi }(x,y)\, \bigr ),\\&\psi _{\chi }^5\ := \forall x \forall y\bigl (\, S_{\alpha }(x,y)\ \leftrightarrow \ E_{\alpha }^{{ Uf}}(x,y)\, \bigr ),\\&\psi _{\chi }^6\ := \forall x \forall y\bigl (\, T_{\alpha }(x,y)\ \leftrightarrow \ E_{\alpha }^{{ Vg}'}(x,y)\, \bigr ). \end{aligned}$$

${Dom}(\chi )$ is either of the sets $\{x\},\ \emptyset$:

$$\begin{aligned}&\psi _{\chi }^1\ := \ \exists x\, S_{\chi }(x)\ \rightarrow \ \exists ^{= 1} x\, E_{\alpha }^{{ Uf}}(x),\\&\psi _{\chi }^2\ := \ \exists x\, E_{\alpha }^{{ Uf}}(x)\ \rightarrow \ \exists x\, S_{\chi }(x),\\&\psi _{\chi }^3\ :=\ \exists x\, T_{\chi }(x) \rightarrow \forall x E_{\alpha }^{{ Vg}'}(x),\\&\psi _{\chi }^4\ := \ \exists x E_{\alpha }^{{ Vg}'}(x)\ \rightarrow \ \exists x\, T_{\chi }(x),\\&\psi _{\chi }^5\ :=\ \forall x \bigl (\, S_{\alpha }(x)\ \leftrightarrow \ E_{\alpha }^{{ Uf}}(x)\, \bigr ),\\&\psi _{\chi }^6\ :=\ \forall x \bigl (\, T_{\alpha }(x)\ \leftrightarrow \ E_{\alpha }^{{ Vg}'}(x)\bigr ). \end{aligned}$$

$${Dom}(\chi )$$ is $$\{y\}$$:

$$\begin{aligned}&\psi _{\chi }^1\ := \ \forall y\bigl (\ S_{\chi }(y)\ \rightarrow \ \exists ^{= 1} x\, E_{\alpha }^{{ Uf}}(x,y)\, \bigr ),\\&\psi _{\chi }^2\ := \ \forall x\forall y\bigl (\, E_{\alpha }^{{ Uf}}(x,y)\ \rightarrow \ S_{\chi }(y)\, \bigr ),\\&\psi _{\chi }^3\ :=\ \forall y\bigl (\, T_{\chi }(y) \rightarrow \forall x E_{\alpha }^{{ Vg}'}(x,y)\bigr ),\\&\psi _{\chi }^4\ := \ \forall x\forall y\bigl (\, E_{\alpha }^{{ Vg}'}(x,y)\ \rightarrow \ T_{\chi }(y)\, \bigr ),\\&\psi _{\chi }^5\ := \forall x \forall y\bigl (\, S_{\alpha }(x,y)\ \leftrightarrow \ E_{\alpha }^{{ Uf}}(x,y)\, \bigr ),\\&\psi _{\chi }^6\ := \forall x \forall y\bigl (\, T_{\alpha }(x,y)\ \leftrightarrow \ E_{\alpha }^{{ Vg}'}(x,y)\, \bigr ). \end{aligned}$$

### Formulae for $\chi = \exists ^{s} y\, \alpha$.

$${Dom}(\chi ) = \{x,y\}$$:

$$\begin{aligned}&\psi _{\chi }^1\ := \ \forall x\forall y\bigl (\ S_{\chi }(x,y)\ \rightarrow \ \exists ^{= 1} y\, E_{\alpha }^{{ Uf}}(x,y)\, \bigr ),\\&\psi _{\chi }^2\ := \ \forall x\forall y\bigl (\, E_{\alpha }^{{ Uf}}(x,y)\ \rightarrow \ \exists y\, S_{\chi }(x,y)\, \bigr ),\\&\psi _{\chi }^3\ :=\ \forall x\forall y\bigl (\, T_{\chi }(x,y) \rightarrow \forall y E_{\alpha }^{{ Vg}'}(x,y)\bigr ),\\&\psi _{\chi }^4\ := \ \forall x\forall y\bigl (\, E_{\alpha }^{{ Vg}'}(x,y)\ \rightarrow \ \exists y\, T_{\chi }(x,y)\, \bigr ),\\&\psi _{\chi }^5\ := \forall x \forall y\bigl (\, S_{\alpha }(x,y)\ \leftrightarrow \ E_{\alpha }^{{ Uf}}(x,y)\, \bigr ),\\&\psi _{\chi }^6\ := \forall x \forall y\bigl (\, T_{\alpha }(x,y)\ \leftrightarrow \ E_{\alpha }^{{ Vg}'}(x,y)\, \bigr ). \end{aligned}$$

If ${Dom}(\chi )$ is either of the sets $\{y\}, \emptyset$, exactly the same formulae $\psi _{\chi }^1,\ldots ,\psi _{\chi }^6$ are used as in the case where $\chi = \exists ^{s} x\, \alpha$ and ${Dom}(\chi )$ is $\{x\}$ or $\emptyset$.

$${Dom}(\chi )$$ is $$\{x\}$$:

$$\begin{aligned}&\psi _{\chi }^1\ := \ \forall y\bigl (\ S_{\chi }(x)\ \rightarrow \ \exists ^{= 1} y\, E_{\alpha }^{{ Uf}}(x,y)\, \bigr ),\\&\psi _{\chi }^2\ := \ \forall x\forall y\bigl (\, E_{\alpha }^{{ Uf}}(x,y)\ \rightarrow \ S_{\chi }(x)\, \bigr ),\\&\psi _{\chi }^3\ :=\ \forall x\forall y\bigl (\, T_{\chi }(x,y) \rightarrow \forall y E_{\alpha }^{{ Vg}'}(x,y)\bigr ),\\&\psi _{\chi }^4\ := \ \forall x\forall y\bigl (\, E_{\alpha }^{{ Vg}'}(x,y)\ \rightarrow \ T_{\chi }(x)\, \bigr ),\\&\psi _{\chi }^5\ :=\ \forall x \forall y\bigl (\, S_{\alpha }(x,y)\ \leftrightarrow \ E_{\alpha }^{{ Uf}}(x,y)\, \bigr ),\\&\psi _{\chi }^6\ :=\ \forall x \forall y\bigl (\, T_{\alpha }(x,y)\ \leftrightarrow \ E_{\alpha }^{{ Vg}'}(x,y)\, \bigr ). \end{aligned}$$

### Formulae for $\chi = \chi _1\vee \chi _2$

$${Dom}(\chi ) = \{x,y\}$$:

$$\begin{aligned}&\psi _{\chi }^1\ := \forall x\forall y \Bigl (\, S_{\chi }(x,y)\ \leftrightarrow \ \bigl (S_{\chi _1}(x,y)\, \vee \, S_{\chi _2}(x,y)\bigr )\Bigr ),\\&\psi _{\chi }^2\ := \forall x\forall y \Bigl (\, T_{\chi }(x,y)\ \leftrightarrow \ T_{\chi _1}(x,y)\Bigr ),\\&\psi _{\chi }^3\ := \forall x\forall y \Bigl (\, T_{\chi }(x,y)\ \leftrightarrow \ T_{\chi _2}(x,y)\Bigr ). \end{aligned}$$

${Dom}(\chi )$ is any of the sets $\{x\}, \{y\}, \emptyset$:

$$\begin{aligned}&\psi _{\chi }^1\ := \forall x \Bigl (\, S_{\chi }(x)\ \leftrightarrow \ \bigl (S_{\chi _1}(x)\, \vee \, S_{\chi _2}(x)\bigr )\Bigr ),\\&\psi _{\chi }^2\ := \forall x \Bigl (\, T_{\chi }(x)\ \leftrightarrow \ T_{\chi _1}(x)\Bigr ),\\&\psi _{\chi }^3\ := \forall x \Bigl (\, T_{\chi }(x)\ \leftrightarrow \ T_{\chi _2}(x)\Bigr ). \end{aligned}$$

### Formulae for $\chi = \lnot \alpha$

$${Dom}(\chi ) = \{x,y\}$$:

$$\begin{aligned}&\psi _{\chi }^1\ := \ \forall x\forall y \bigl (\ S_{\chi }(x,y)\ \leftrightarrow \ T_{\alpha }(x,y)\, \bigr ),\\&\psi _{\chi }^2\ := \ \forall x\forall y\bigl (\ T_{\chi }(x,y)\ \leftrightarrow \ S_{\alpha }(x,y)\, \bigr ). \end{aligned}$$

${Dom}(\chi )$ is any of the sets $\{x\}, \{y\},\emptyset$:

$$\begin{aligned}&\psi _{\chi }^1\ := \ \forall x\bigl (\ S_{\chi }(x)\ \leftrightarrow \ T_{\alpha }(x)\, \bigr ),\\&\psi _{\chi }^2\ := \ \forall x\bigl (\ T_{\chi }(x)\ \leftrightarrow \ S_{\alpha }(x)\, \bigr ). \end{aligned}$$

### $\chi$ is an Atomic Formula

$\chi$ is a first-order atom and ${Dom}(\chi ) = \{x,y\}$:

$$\begin{aligned}&\psi _{\chi }^1\ :=\ \forall x \forall y\bigl (\, S_{\chi }(x,y)\rightarrow \chi \, \bigr ),\\&\psi _{\chi }^2\ :=\ \forall x \forall y\bigl (\, T_{\chi }(x,y)\rightarrow \lnot \chi \, \bigr ). \end{aligned}$$

$\chi$ is a first-order atom and ${Dom}(\chi )$ is $\{x\}$:

$$\begin{aligned}&\psi _{\chi }^1\ :=\ \forall x \bigl (\, S_{\chi }(x)\rightarrow \chi \, \bigr ),\\&\psi _{\chi }^2\ :=\ \forall x \bigl (\, T_{\chi }(x)\rightarrow \lnot \chi \, \bigr ). \end{aligned}$$

$\chi$ is a first-order atom and ${Dom}(\chi )$ is $\{y\}$:

$$\begin{aligned}&\psi _{\chi }^1\ :=\ \forall y \bigl (\, S_{\chi }(y)\rightarrow \chi \, \bigr ),\\&\psi _{\chi }^2\ :=\ \forall y \bigl (\, T_{\chi }(y)\rightarrow \lnot \chi \, \bigr ). \end{aligned}$$

If $$\chi $$ is the formula $$=\!\!(x,y)$$, then $${Dom}(\chi ) = \{x,y\}$$. We define $$\psi _{\chi }^1\ :=\ \lnot \exists x\exists ^{\ge 2}y\,$$$$ S_{\chi }(x,y)$$ and $$\psi _{\chi }^2\ :=\ \lnot \exists x\exists y\, T_{\chi }(x,y)$$. If $$\chi $$ is the formula $$=\!\!(y,x)$$, then $${Dom}(\chi ) = \{x,y\}$$. We define $$\psi _{\chi }^1\ :=\ \lnot \exists y\exists ^{\ge 2} x\, S_{\chi }(x,y)$$ and $$\psi _{\chi }^2\ :=\ \lnot \exists x\exists y\, T_{\chi }(x,y)$$.

If $$\chi $$ is the formula $$=\!\!(x)$$ and $${Dom}(\chi ) = \{x,y\}$$, we define $$\psi _{\chi }^1\ :=\ \lnot \exists ^{\ge 2} x\, \exists y$$$$\, S_{\chi }(x,y)$$ and $$\psi _{\chi }^2\ :=\ \lnot \exists x\exists y\, T_{\chi }(x,y)$$. If $$\chi $$ is the formula $$=\!\!(x)$$ and $${Dom}(\chi ) = \{x\}$$, we define $$\psi _{\chi }^1\ :=\ \lnot \exists ^{\ge 2} x\, S_{\chi }(x)$$ and $$\psi _{\chi }^2\ :=\ \lnot \exists x\, T_{\chi }(x)$$.

If $$\chi $$ is the formula $$=\!\!(y)$$ and $${Dom}(\chi ) = \{x,y\}$$, we define $$\psi _{\chi }^1\ :=\ \lnot \exists ^{\ge 2} y\, \exists x\, S_{\chi }$$$$(x,y)$$ and $$\psi _{\chi }^2\ :=\ \lnot \exists x\exists y\, T_{\chi }(x,y)$$. If $$\chi $$ is the formula $$=\!\!(y)$$ and $${Dom}(\chi ) = \{y\}$$, we define $$\psi _{\chi }^1\ :=\ \lnot \exists ^{\ge 2} x\, S_{\chi }(x)$$ and $$\psi _{\chi }^2\ :=\ \lnot \exists x\, T_{\chi }(x)$$.

## Footnotes

1 Assignments are functions that give values to variables in the domain of the model under consideration.

2 Strictly speaking, if the valuations in *X* already give an interpretation to *x*, then the team *X* is *modified* by altering the assignments rather than extending them.

3 In some cases non-first-order atoms cannot be negated at all.

4 Intuition only!.

5 The formula $\varphi$ can indeed belong to any extension of first-order logic with ordinary generalized quantifiers, minor quantifiers, and generalized atoms.

6 Consider the connective $\vee ^s$ defined in Sect. 4. We can add this connective into the language considered. The rules for a position $(\mathfrak {A},t,+,\psi \vee ^s \psi ')$ are exactly as for $(\mathfrak {A},t,+,\psi \vee \psi ')$, but with the exception that the choice $\{\psi ,\psi '\}$ by $\fancyscript{A}$ is not allowed. The rules for a position $(\mathfrak {A},t,-,\psi \vee ^s\psi ')$ are the same as for a position $(\mathfrak {A},t,-,\psi \vee \psi ')$. As the reader can easily check, Theorem 1 below goes through even when the language is extended by $\vee ^s$. We could consider further connectives and even define a natural notion of a *minor connective*, but we shall not do that for the sake of brevity.

7 We of course recall that this is nothing more than the background intuition.

8 Of course Tarski’s semantics also ties truth of first-order formulae to the notion of a model, and additionally provides an inductive method for computing truth values of formulae based on the truth values of atoms.

9 Of course game-theoretic truth definitions are still usually *described* in natural language.

10 It turns out that there is no need for symbols $E_{\alpha }^{{ Uf}'}$, $E^{ Vg}$. In fact, even the symbols $E_{\alpha }^{{ Uf}}$ and $E_{\alpha }^{{ Vg}'}$ could be eliminated, but we keep them for the sake of presentation. The reader may consider further minor quantifiers for which the proofs in this section go trough. In doing so, using extra predicates $E_{\alpha }^{{ Uf}},\, E_{\alpha }^{{ Vg}'},\, E_{\alpha }^{{ Uf}'},E^{ Vg}$ may help. By analyzing our proof below, it is easy to see that for example the total existential quantifier $\exists ^t$ could be added to $\hbox {DC}^2$ without sacrificing $\hbox {NEXPTIME}$-completeness.
